# MYCN and SNRPD3 cooperate to maintain a balance of alternative splicing events that drives neuroblastoma progression

**DOI:** 10.1038/s41388-023-02897-y

**Published:** 2023-12-04

**Authors:** Alice Salib, Nisitha Jayatilleke, Janith A. Seneviratne, Chelsea Mayoh, Katleen De Preter, Frank Speleman, Belamy B. Cheung, Daniel R. Carter, Glenn M. Marshall

**Affiliations:** 1 Children’s Cancer Institute, Lowy Cancer Research Centre, UNSW Sydney, Kensington, NSW 2052 Australia; 2 School of Women’s and Children’s Health, UNSW Sydney, Kensington, NSW 2052 Australia; 3 Center for Medical Genetics (CMGG), Ghent University, Medical Research Building (MRB1), Ghent, Belgium; 4 School of Biomedical Engineering, University of Technology Sydney, Ultimo, NSW 2007 Australia; 5 Kids Cancer Centre, Sydney Children’s Hospital, Randwick, NSW 2031 Australia

**Keywords:** Paediatric cancer, Cancer genomics

## Abstract

Many of the pro-tumorigenic functions of the oncogene *MYCN* are attributed to its regulation of global gene expression programs. Alternative splicing is another important regulator of gene expression and has been implicated in neuroblastoma development, however, the molecular mechanisms remain unknown. We found that MYCN up-regulated the expression of the core spliceosomal protein, SNRPD3, in models of neuroblastoma initiation and progression. High mRNA expression of *SNRPD3* in human neuroblastoma tissues was a strong, independent prognostic factor for poor patient outcome. Repression of *SNRPD3* expression correlated with loss of colony formation in vitro and reduced tumorigenicity in vivo. The effect of SNRPD3 on cell viability was in part dependent on MYCN as an oncogenic co-factor. RNA-sequencing revealed a global increase in the number of genes being differentially spliced when MYCN was overexpressed. Surprisingly, depletion of SNRPD3 in the presence of overexpressed MYCN further increased differential splicing, particularly of cell cycle regulators, such as BIRC5 and CDK10. MYCN directly bound SNRPD3, and the protein arginine methyltransferase, PRMT5, consequently increasing SNRPD3 methylation. Indeed, the PRMT5 inhibitor, JNJ-64619178, reduced cell viability and SNRPD3 methylation in neuroblastoma cells with high SNRPD3 and MYCN expression. Our findings demonstrate a functional relationship between MYCN and SNRPD3, which maintains the fidelity of MYCN-driven alternative splicing in the narrow range required for neuroblastoma cell growth. SNRPD3 methylation and its protein-protein interface with MYCN represent novel therapeutic targets.

**Figure:**
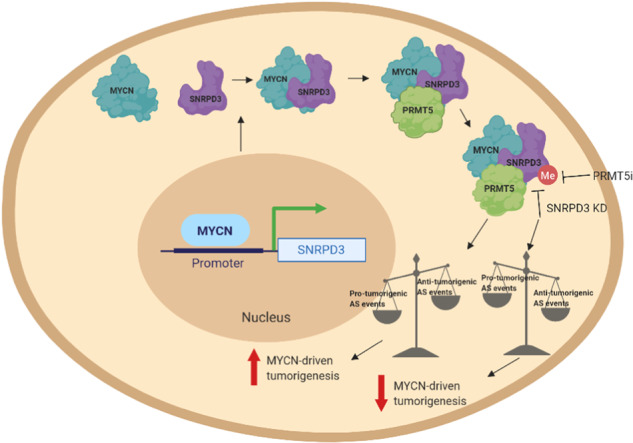
Hypothetical model for SNRPD3 as a co-factor for MYCN oncogenesis. SNRPD3 and MYCN participate in a regulatory loop to balance splicing fidelity in neuroblastoma cells. First MYCN transactivates SNRPD3 to lead to high-level expression. Second, SNRPD3 and MYCN form a protein complex involving PRMT5. Third, this leads to balanced alterative splicing (AS) activitiy that is favorable to neuroblastoma. Together this forms as a therapeutic vulnerability where SNRPD3 perturbation or PRMT5 inhibitors are selectively toxic to neuroblastoma by conditionally disturbing splicing activity.

Hypothetical model for SNRPD3 as a co-factor for MYCN oncogenesis. SNRPD3 and MYCN participate in a regulatory loop to balance splicing fidelity in neuroblastoma cells. First MYCN transactivates SNRPD3 to lead to high-level expression. Second, SNRPD3 and MYCN form a protein complex involving PRMT5. Third, this leads to balanced alterative splicing (AS) activitiy that is favorable to neuroblastoma. Together this forms as a therapeutic vulnerability where SNRPD3 perturbation or PRMT5 inhibitors are selectively toxic to neuroblastoma by conditionally disturbing splicing activity.

## Introduction

Neuroblastoma is a common solid tumour in early childhood with only a 50% survival rate for high-risk patients [1]. Amplification of the MYCN oncogene remains the single most important genetic predictor of poor patient prognosis [1]. While MYCN would make an ideal target, it is extremely difficult to target due to its protein structure having few suitable docking sites for small molecule inhibitors [2]. Therefore, it is important to explore MYCN co-factors as well as pathways that are altered because of *MYCN* amplification as potential therapeutic targets. Many of the pro-tumorigenic functions of MYCN are attributed to its ability to regulate global gene expression programs [3–5]. Alternative splicing is another important regulator of gene expression in normal or malignant cells and is aberrantly regulated in cancer cells to produce cancer-specific isoforms that drive cancer progression by promoting growth and survival advantages [6–9]. Several different mechanisms can lead to aberrant splicing, which include: alterations in cancer-related genes that directly affect their pre-mRNA splicing; mutations in genes encoding snRNAs, splicing factors or core spliceosomal subunits; and disruptions in the balance of the expression of RNA-binding proteins [10].

Effective alternative splicing depends upon an intact and highly coordinated process carried out by the spliceosome. The spliceosome is a dynamic machinery composed of several different components, which can contain over 300 proteins [11]. However, a core component of this complex is a group of seven proteins referred to as Sm proteins including the Small Nuclear Ribonucleoprotein D3 Polypeptide (SNRPD3). These seven Sm proteins result in the formation of a ring structure that is then incorporated into the spliceosome responsible for its stability and nuclear import [12, 13]. The ring is assembled when the 3 Sm proteins, SNRPB, SNRPD1, and SNRPD3 are symmetrically dimethylated by the protein arginine methyltransferase, PRMT5 at the arginine/glycine-rich “RG motifs” in the region carboxyl-terminal to their Sm domains [14, 15]. Interestingly, a recent study has proposed that the core Sm proteins are cancer-selective lethal targets in non-small cell lung cancer, compared to the more commonly mutated splicing factor, SF3B1, where loss of function is toxic to non-malignant cells [16]. The role of SNRPD3 in cancer has really only been discovered in the last decade [17]. Overexpression of SNRPD3 has been observed in several cancers but has not yet been linked to childhood cancer [18–21].

Alternative splicing has been shown to be involved in neuroblastoma tumorigenesis [22–24]. Moreover, it is known that *MYCN*-amplified neuroblastoma tumours have a distinct alternative splicing pattern and exhibit greater differential splicing [23, 24]. However, no direct involvement of the spliceosome has been described in neuroblastoma, and thus we still do not understand the regulatory mechanisms that underpin MYCN-driven alternative splicing changes. There is growing evidence that MYCN up-regulates components of the spliceosome to drive splicing events, which promote MYCN-driven tumorigenesis [23–26]. Along with MYCN, other proto-oncogenes such as MYC and KRAS up-regulate components of the spliceosome in cancer, indicating a possible addiction to a hyperactivated spliceosome, and a potential novel therapeutic target [21, 27, 28].

Here we show for the first time that SNRPD3 is directly up-regulated by MYCN and together these two factors form a protein complex with the protein arginine methyltransferase, PRMT5. High SNRPD3 expression is correlated with poor patient outcome. We found that SNRPD3 ensures the fidelity and balance of MYCN-driven alternative splicing events required for neuroblastoma oncogenesis. Our findings highlight a potential new therapeutic approach for the treatment of high-risk neuroblastoma.

## Results

### Identification of *SNRPD3*, a core spliceosome assembly gene, as a prognostic factor in neuroblastoma

**Fig. 1:**
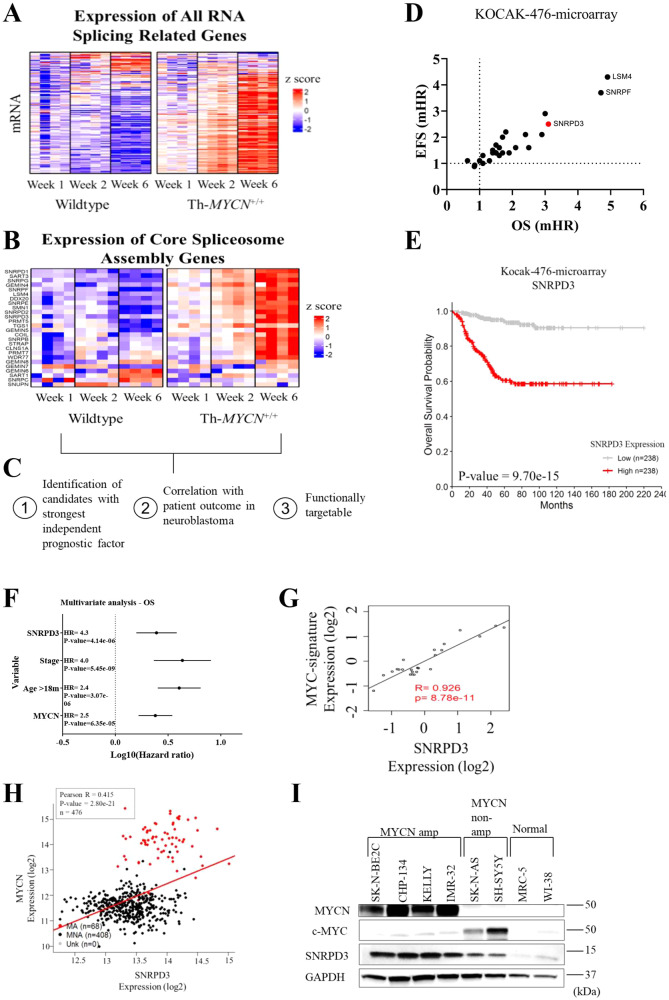
Some core snRNP assembly genes are up-regulated in neuroblastoma and are prognostic for patient outcomes. A heat map of a mRNA microarray analysis on ganglia cells collected from transgenic Th-MYCN ^+ /+^ and wildtype mice at 1, 2, and 6 weeks of age assessing the expression of (**A**) all RNA splicing-related genes, or (**B**) the core spliceosome assembly genes. **C** All 26 core spliceosome assembly genes (as defined by the GO term, GO:0000387) were assessed based on the three given criteria to identify possible candidate genes. **D** Overall (OS) and event-free survival (EFS) multivariate CoxPH analysis using the Kocak (*n* = 476) neuroblastoma patient cohort was conducted on the core spliceosome assembly genes (as defined by the GO term, GO:0000387). The dot plot represents the median hazard ratios (mHR) from the CoxPH models for each gene regarding EFS and OS. **E** Kaplan–Meier OS curve obtained from Kocak neuroblastoma patient cohort (*n* = 476) dichotomised on median SNRPD3 expression. **F** OS multivariate CoxPH analysis on Kocak cohort (*n* = 649) with SNRPD3 gene expression against classic neuroblastoma prognostic factors, dots represent the median HR (hazard ratio) whilst lines represent the 95% confidence interval. **G** Scatter plot with a linear regression fit of mRNA expression levels from the Th-*MYCN*^+/+^ mouse tissues for *MYC*-signature vs *SNRPD3* gene expression (6 weeks of age, microarray, log2). **H** Scatter plot with linear regression fit for the Kocak neuroblastoma patient cohort for *MYCN* vs *SNRPD3* gene expression (mRNA microarray, log2 expression). **I** Western blot analysis for SNRPD3, cMYC and MYCN expression in a range of *MYCN*-amplified (SK-N-BE(2)-C, CHP-134, KELLY, IMR-32), MYCN non-amplified (SK-N-AS, SH-SY5Y), and normal lung fibroblasts (MRC-5, WI-38) cells. GAPDH is a protein loading control. Western blot is a representative image of *n* = 3 independent experiments.

To understand the regulation of alternatively spliced genes during MYCN-driven neuroblastoma initiation and progression, ganglia and tumour tissue were collected at 1, 2, and 6 weeks of age from transgenic homozygote Th-*MYCN*^+/+^ and wildtype littermate mice, as previously described [29]. All homozygote Th-*MYCN*^+/+^ transgenic mice develop neuroblastoma in the sympathetic ganglia at 6−7 weeks of age, after a precancer stage of neuroblast hyperplasia in the ganglia during the first 2 weeks of life [30]. Microarray analysis revealed a near global upregulation of most of the 300 genes involved in RNA splicing during neuroblastoma progression in Th-*MYCN*^+/+^ mice (Fig. 1A). Further analysis of the 26 core spliceosome assembly genes (as defined by the GO term, GO:0000387), also revealed strong upregulation in Th-*MYCN*^+/+^ mice compared with wildtype mice, which was most marked at the late stages of tumour progression (Fig. 1B). To identify possible candidate genes that are important for neuroblastoma progression, the 26 core spliceosome assembly genes were assessed against the following three criteria: strong independent prognostic factor, association with patient outcome in neuroblastoma, and functionally targetable (Fig. 1C). Microarray data from the Kocak (*n* = 649) and SEQC (*n* = 498) neuroblastoma patient cohorts were utilised to analyse the 26 genes that make up the core spliceosome assembly genes (GO:0000387). We found that SNRPD3, SNRPF and LSM4 had the strongest independent prognostic effect on neuroblastoma patient outcome among the core spliceosome assembly genes as measured by hazard ratios for event-free (EFS) and overall (OS) patient survival (Fig. 1D and Supplementary Fig. 1A). SNRPD3 was selected for further study due to the availability of known indirect inhibitors in phase I and II clinical trials, JNJ-64619178 and GSK3326595 (ClinicalTrials.gov identifier: NCT03573310, NCT03614728, and NCT02783300). In the Kocak dataset, high SNRPD3 expression significantly correlated with poor patient outcome (Fig. 1E and Supplementary Fig. 1B). Furthermore, multivariate Cox regression analysis for both OS and EFS showed *SNRPD3* expression to be an independent prognostic factor when compared to well established clinical prognostic factors such as INSS clinical stage (4 vs other), age at diagnosis (>18 months vs <18 months), and *MYCN* amplification status (amplified vs non-amplified) (Fig. 1F and Supplementary Fig. 1C). Patients with poor prognostic factors: Stage 4 disease (*p* = 4.0e-15), those diagnosed at greater than 18 months of age (*p* = 2.3e-06), or with *MYCN* amplification (*p* = 9.5e-14), all had significantly higher *SNRPD3* gene expression (Supplementary Fig. 1D). These findings were also observed in the independent SEQC neuroblastoma patient tumour dataset (Supplementary Fig. 1E−G). Next, we examined the correlation between *SNRPD3* and *MYCN* mRNA during tumorigenesis in the Th-*MYCN*^+/+^ mouse ganglia tissue. Microarray mRNA expression analysis revealed increasing *SNRPD3* expression during tumorigenesis, synchronous with a previously described MYC-signature of target gene expression shared with MYCN [29] (Supplementary Fig. 1H). Importantly, *SNRPD3* expression strongly correlated (*R* = 0.926) with the level of MYC-signature expression during neuroblastoma (Fig. 1G). In the Kocak and SEQC datasets *SNRPD3* expression also significantly correlated with MYCN expression (Fig. 1H and Supplementary Fig. 1I). Lastly, we found that *MYCN*-amplified (SK-N-BE(2)-C, CHP-134, KELLY and IMR-32) human neuroblastoma cell lines had significantly higher SNRPD3 expression when compared to MYCN non-amplified cell lines (SK-N-AS and SH-SY5Y) and human fibroblast (MRC-5 and WI-38) cell lines (Fig. 1I and Supplementary Fig. 1J). Taken together these results indicate that SNRPD3 may have a functional relationship with MYCN and has an important role in neuroblastoma tumorigenesis.

### SNRPD3 is a MYCN transcriptional target and is required for MYCN-driven cell viability

**Fig. 2:**
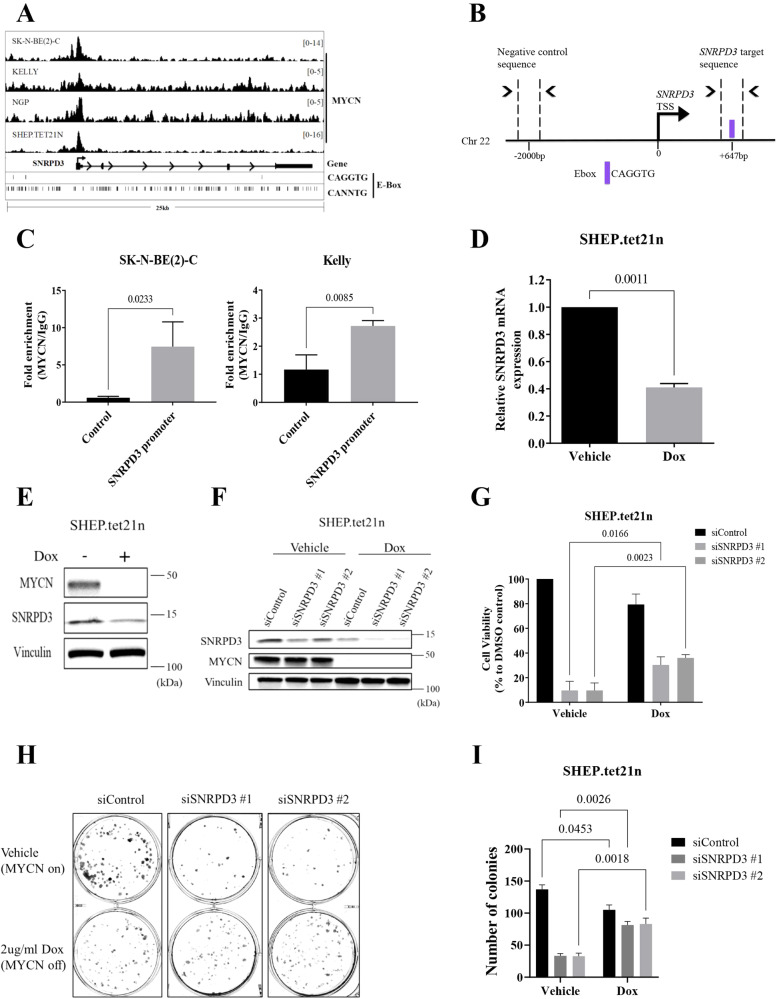
MYCN regulates the expression of the *SNRPD3* gene in neuroblastoma cells. **A** ChIP-sequencing traces from publicly available databases [3] for MYCN binding the SNRPD3 gene in a panel of MYCN expressing neuroblastoma cells detailing MYCN binding motifs at canonical (CACGTG) and non-canonical (CANNTG) E-boxes **B** Schematic representation of the *SNRPD3* target sequence (+647 bp from SNRPD3 TSS) and negative control target sequence (−2000 bp from SNRPD3 TSS) used for quantitative real time PCR (QPCR) following chromatin-immunoprecipitation (ChIP), detailing the MYCN peak summit and its distance from TSS. **C** ChIP-qPCR assay for the negative control region or the SNRPD3 promoter containing the MYCN binding site in SK-N-BE(2)-C and KELLY cells. Results represent *n* = 3 independent biological replicates, mean ± SEM. *P*-values were determined by two-tailed *t* test comparing control against SNRPD3 promoter. **D** SNRPD3 mRNA and **E** protein expression measured in SHEP.tet21n cells treated with doxycycline (Dox; MYCN off) or without Dox (Vehicle; MYCN on). Results represent *n* = 3 independent biological replicates, mean ± SEM, where *P* values were determined by two-tailed *t* test comparing vehicle against Dox. **F** A representative immunoblot of MYCN and SNRPD3 expression in SHEP.tet21n cells treated with either vehicle or Dox following SNRPD3 siRNA knockdown. **G** Cell viability was measured 72 h after SHEP.tet21n cells treated with either vehicle (DMSO) or Dox were transfected with control siRNA, or the two SNRPD3 siRNAs. Results represent *n* = 3 independent biological replicates, mean ± SEM, where the *P* value was determined through a two-way ANOVA, with Tukey’s multiple comparison tests. **H** SHEP.tet21n cells treated with either vehicle or Dox were transfected with control siRNA or the two SNRPD3 siRNAs for 10 days, followed by colony formation assays. Differences in colony formation were compared to siControl and **I** number of colonies quantified. Results represent *n* = 3 independent biological replicates, mean ± SEM, where the *P* value was determined through a two-way ANOVA, with Tukey’s multiple comparison tests.

As *SNRPD3* expression was highly expressed in *MYCN*-amplified cell lines, we hypothesised that *SNRPD3* is a MYCN transcriptional target gene. Publicly available ChIP-sequencing data [3] revealed that MYCN bound to the *SNRPD3* promoter (Fig. 2A). To validate MYCN binding to the promoter of SNRPD3 we performed Chromatin Immunoprecipitation (ChIP) with anti-MYCN and IgG antibodies followed by quantitative real time PCR (RT-qPCR) using primers that target the CAGGTG E-box ( + 647 bp upstream of the SNRPD3 transcription start site (TSS)) and a negative control (Control; −2000 bp downstream of SNRPD3 TSS) region in the *MYCN*-amplified human neuroblastoma SK-N-BE(2)-C and KELLY cell lines (Fig. 2B). We found that MYCN occupancy at the *SNRPD3* promoter was 7-fold higher than the negative control region in SK-N-BE(2)-C cells and 2.5-fold higher in KELLY cells (Fig. 2C). Furthermore, SNRPD3 mRNA (Fig. 2D) and protein (Fig. 2E) expression was significantly reduced when *MYCN* was switched off by the addition of doxycycline (Dox) to the SHEP.tet21n cell line, which carries a Dox-inducible MYCN knockdown. Together these results demonstrate that *SNRPD3* transcription is regulated by MYCN. The functional relationship between MYCN and SNRPD3 was further examined using SHEP.tet21n cells, transfected with SNRPD3 siRNA. The suppression of SNRPD3 resulted in a marked reduction of cell viability and colony formation in the presence of MYCN with a lesser reduction in viability and colony formation when MYCN was switched off (Fig. 2F−I). These findings suggest that in the presence of MYCN, SNRPD3 has profound effects on cell viability and proliferation, which are, in part, reduced in the absence of MYCN. However, these data, and the independent prognostic value of SNRPD3 expression in human neuroblastoma, indicate the two proteins affect the malignant phenotype by both dependent and independent mechanisms.

### SNRPD3 is required for the survival and proliferation of *MYCN*-amplified neuroblastoma cells

**Fig. 3:**
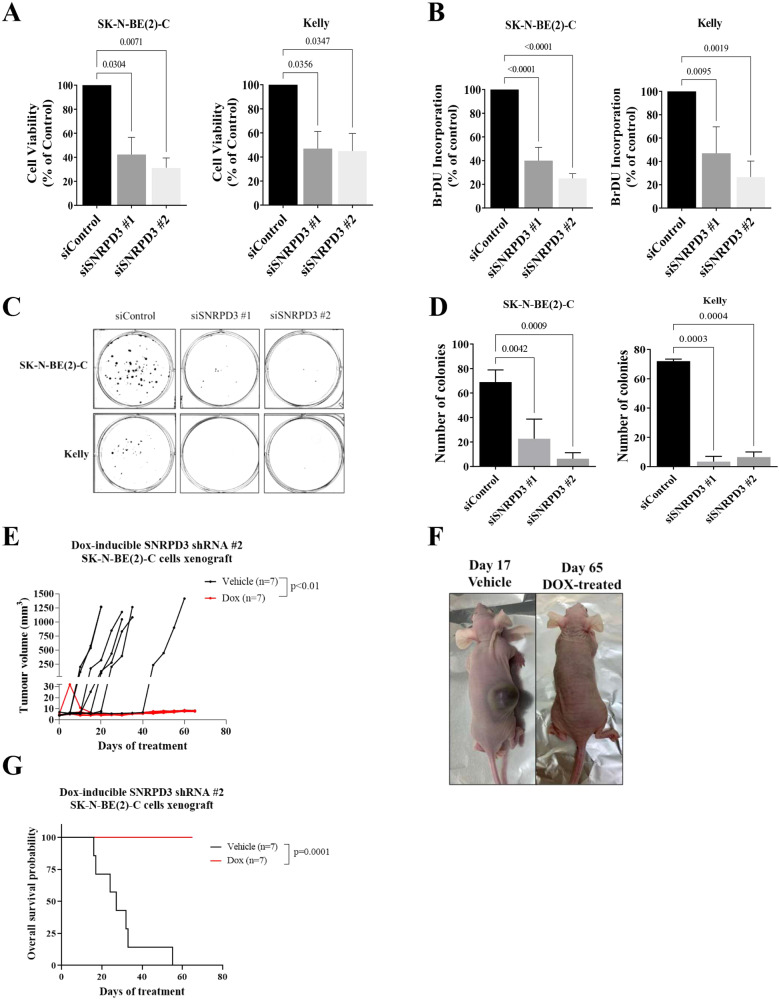
SNRPD3 is required for the growth and proliferation of *MYCN*-amplified neuroblastoma cells. SK-N-BE(2)-C and KELLY cells were transfected with siRNAs targeted against SNRPD3 or a control siRNA for 72 h, then subjected to (**A**) cell viability and (**B**) cell proliferation measurements. Differences in cell viability and proliferation were compared with siControl. **C** SK-N-BE(2)-C and KELLY cells were transfected with siRNAs targeting SNRPD3 or a control siRNA for 10 days (SK-N-BE(2)-C) or 14 days (KELLY), followed by colony formation assays. Differences in colony formation were compared to siControl and (**D**) the number of colonies were quantified. This is a representative image of three biologically independent experiments (*n* = 3), mean ± SEM, where the *P* value was determined through a one-way ANOVA, with Dunnett multiple comparison testing. SK-N-BE(2)-C cells expressing SNRPD3 shRNA (shSNRPD3) were xenografted into immunodeficient nude mice. Once tumours reached 4−5 mm, mice were divided into DOX (2 mg/mL doxycycline) or vehicle (water containing 5% sucrose) control (Vehicle) subgroups. **E** Tumour volume was measured (*n* = 7 mice per treatment group) from day 0 post-treatment until the tumour reached ≥1000 mm^3^ or a maximum holding time of 12 weeks. The effect of DOX on tumour progression was evaluated using a two-way ANOVA. **F** Representative photos of either vehicle- (day 17 post treatment) or DOX-treated (day 65 post treatment) mice. **G** Kaplan–Meier survival curves showed the probability of overall survival of the mice (*n* = 7 mice per treatment group). A log rank test was used to obtain *p*-value. All results represent *n* = 3 independent biological replicates, mean ± SEM, where the *P* value was determined through a one-way ANOVA, with Dunnett multiple comparison testing, unless stated otherwise.

To investigate whether SNRPD3 plays a role in the survival and proliferation of *MYCN*-amplified neuroblastoma, in vitro, phenotypic assays and in vivo xenograft experiments were performed. Depletion of *SNRPD3* using two siRNAs targeting SNRPD3 in the *MYCN*-amplified SK-N-BE(2)-C and KELLY cell lines caused a significant reduction in cell viability and cell proliferation, as measured by alamar blue and BrdU incorporation assays, respectively (Fig. 3A, B). Similarly, clonogenic assays in SK-N-BE(2)-C and KELLY cells demonstrated that siRNA-mediated knockdown of *SNRPD3* led to almost complete abrogation of clonogenicity (Fig. 3C, D and Supplementary Fig. 2A). Given that we observed MYCN-dependent and independent effects of SNRPD3 on cell viability and proliferation, we performed siRNA knockdowns of SNRPD3 in two *MYCN* non-amplified cell lines, SH-SY5Y, which has high MYC expression and SK-N-FI, which has no to low MYCN and MYC expression. We found that depletion of SNRPD3 resulted in a decrease in cell viability in SH-SY5Y but not in SK-N-FI cells (Supplementary Fig. 2B). To assess the in vivo impact of SNRPD3 depletion in neuroblastoma, pooled Dox-inducible control shRNA (shControl) or two SNRPD3 shRNA (shSNRPD3 #1 and #2) SK-N-BE(2)-C cell lines were generated. Suppression of SNRPD3, through the addition of Dox resulted in a significant reduction in colony formation and cell viability, similar to what was observed for the siRNA (Supplementary Fig. 2C−F). We next examined the effects of SNRPD3 on neuroblastoma cell growth in xenograft mouse modelling. Four- to five-week-old Balb/c nude mice were subcutaneously injected with the Dox-inducible SNRPD3 shRNA SK-N-BE(2)-C cells. When tumours reached 4−5 mm, mice were divided into Dox- or vehicle control-treated groups and given 5% sucrose water supplemented with or without 2 mg/ml Dox, until the tumours reached 1000 mm^3^ or maximum holding time of 12 weeks. Treatment with Dox to supress SNRPD3 expression completely ablated neuroblastoma tumorigenesis in this xenograft model and resulted in 100% survival (Fig. 3E−G). Indicating that SNRPD3 plays a vital role in neuroblastoma tumorigenesis.

### SNRPD3 maintains the balance of MYCN-driven alternative splicing events required for neuroblastoma tumorigenesis

**Fig. 4:**
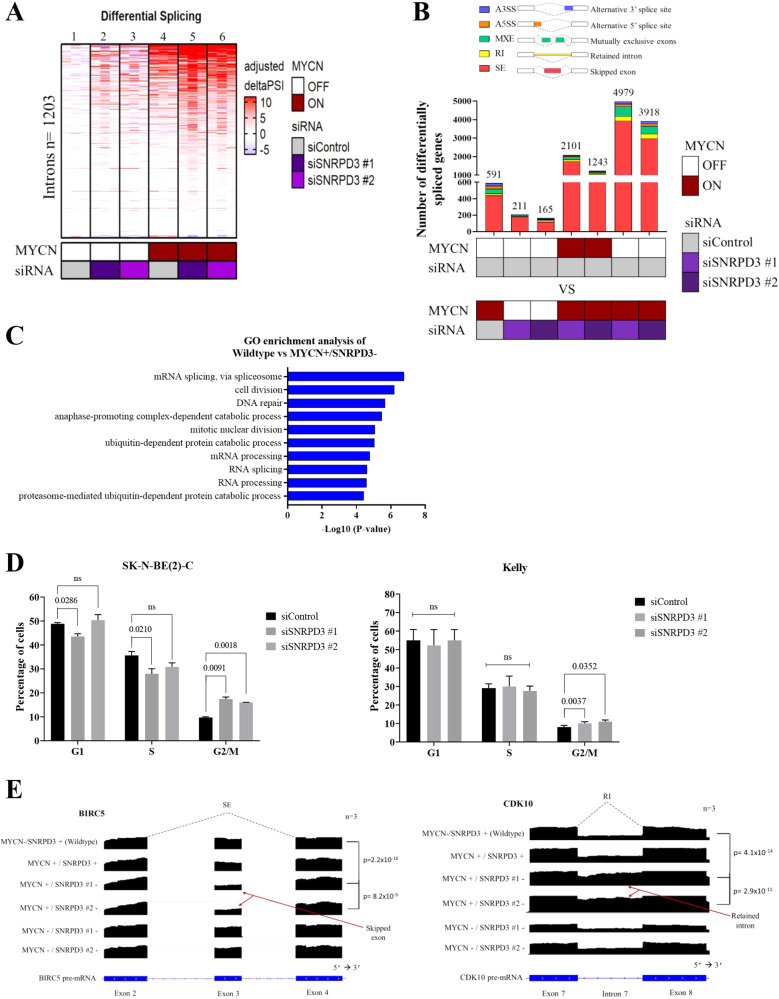
SNRPD3 alters the splicing of cell cycle genes in a MYCN-dependant manner. **A** Heat map showing leafcutter analysis of differential splicing from SHEP.tet21n cells, treated with (to repress MYCN) or without Dox (to induce MYCN), followed by depletion of SNRPD3 with two siRNAs or a control siRNA (siControl). Leafcutter calculated the delta PSI (percentage spliced in) of the *n* = 1203 introns that were found to be differentially spliced in at least 1 pair of samples using MYCN OFF/siControl condition (lane 1) as a reference. **B** The number of significant differentially spliced genes as determined by rMATS analysis in SHEP.tet21n cells treated with (MYCN off) or without (MYCN on) Dox and transfected with either control or SNRPD3 siRNA. Skipped exon (SE), Retained intron (RI), Mutually exclusive exon (MXE), Alternative 5’ splice site (A5SS), Alternative 3’ splice site (A3SS). **C** Gene ontology showing enriched pathways in SHEP.tet21n cells treated with Dox (to repress MYCN) and transfected with control siRNA (wildtype; MYCN-/SNRPD3 + ) compared to SHEP.tet21n cells treated without Dox (to induce MYCN) and transfected with SNRPD3 siRNA (MYCN + /SNRPD3-). **D** Flow cytometry analysis of cell cycle distribution in SK-N-BE(2)-C and KELLY cells transfected with SNRPD3 siRNAs for 72 h followed by propidium iodide (PI) treatment. *P*-values were determined by two-sided *t* test comparing control siRNA against siSNRPD3 #1 or siSNRPD3 #2. **E** RNA-seq coverage plots for *BIRC5* and *CDK10* and the corresponding alternative splicing event. Arrows point to which exon is skipped or which intron is retained. Skipped exon (SE), Retained intron (RI). All results represent *n* = 3 independent biological replicates.

Given the large number of genes alternatively spliced in high-risk neuroblastoma patients [23], we next sought to investigate the changes in the splicing landscape resulting from disruptions to MYCN and SNRPD3 expression. SHEP.tet21n cells treated with vehicle (MYCN + ) or Dox (MYCN-) were additionally transfected with control (SNRPD3 +) or SNRPD3 (SNRPD3-) siRNA, followed by RNA-seq. Therefore, the following nomenclature will be used to represent each sample condition; MYCN-/SNRPD3+ (wildtype), MYCN + /SNRPD3 + , MYCN + /SNRPD3-, and MYCN- /SNRPD3-. Consistent with previous reports showing MYC proteins induce greater differential splicing when dysregulated in cancer cells [28, 31], our analysis using the leafcutter program [32] revealed that neuroblastoma cells overexpressing MYCN exhibited a greater number of differentially spliced genes compared to cells expressing no MYCN (lane 4 vs lane 1, Fig. 4A). Surprisingly, SNRPD3 knockdown in MYCN over-expressing cells resulted in a further increase in the number of genes being differentially spliced (lanes 5–6 vs lane 4, Fig. 4A) suggesting that SNRPD3 maintained the fidelity of specific alternative splicing events required by MYCN. This pronounced difference in splicing was MYCN-specific, with SNRPD3 knockdown alone showing fewer effects on differential splicing (lane 2–3 vs lane 1, Fig. 4A).

Next, rMATS analysis [33] was performed on the RNA-seq dataset to identify the alternative splicing events that take place. Multiple comparisons were made to assess the effect of MYCN alone on splicing (wildtype vs MYCN + /SNRPD3 + ), the effect of SNRPD3 alone on splicing (wildtype vs MYCN-/SNRPD3-), the effect of SNRPD3 on splicing in the context of MYCN overexpression (MYCN + /SNRPD3+ vs MYCN + /SNRPD3-), and lastly, the effect of MYCN on splicing in the context of SNRPD3 depletion (wildtype vs MYCN + /SNRPD3-). The predominant splicing event to take place among all comparisons was exon skipping, however, an increase in intron retention and mutually exclusive exon usage was observed in the wildtype vs MYCN + /SNRPD3- comparison (Fig. 4B). Gene ontology (GO) enrichment analysis was performed on the gene sets identified from the rMATS analysis that were statistically significant (*p* < 0.05). In the wildtype vs MYCN + /SNRPD3- comparison, we found enrichment of splicing regulation, cell cycle and DNA damage pathways (Fig. 4C). This observation was unique to the wildtype vs MYCN + /SNRPD3- comparison (Fig. 4C), where depletion of SNRPD3 in the context of MYCN overexpression (MYCN + /SNRPD3+ vs MYCN + /SNRPD3-) (Supplementary Fig. 3A) saw enrichment of splicing events of predominantly splicing regulatory factors including symmetric dimethylarginine. This observation demonstrated that disruption to a core splicing factor, SNRPD3 in the presence of the oncogenic driver MYCN, resulted in comprehensive changes to RNA splicing. Importantly, the effects of SNRPD3 on the splicing landscape under the MYCN driver gene (Supplementary Fig. 3A) differs from the effects of SNRPD3-alone (Supplementary Fig. 3B), where SNRPD3 predominantly enriched pathways involved in immune and DNA damage pathways. Overexpression of MYCN-alone predominately enriched apoptotic pathways (Supplementary Fig. 3C). These results suggests that SNRPD3 specifically maintains splicing fidelity of MYCN-related mis-splicing events in genes responsible for cell cycle and DNA damage pathways. To understand the effects of SNRPD3 knockdown on cell cycle progression, we first performed flow cytometry analysis. We found that suppression of SNRPD3 caused an increase in the proportion of neuroblastoma cells in the G_2_/M cell cycle phase resulting in G_2_/M arrest (Fig. 4D).

We identified *BIRC5* (Survivin) and *CDK10*, to be the most significantly differentially spliced cell cycle genes in our RNA-seq dataset when MYCN is overexpressed and SNRPD3 is repressed (MYCN + /SNRPD3-) (Supplementary Table 2). Our analysis revealed exon 3 skipping for BIRC5 and intron 7 retention for CDK10 in MYCN + /SNRPD3- cells compared to wildtype cells (Fig. 4E). Our RNA-seq data showed that BIRC5 exon 3 skipping and CDK10 intron 7 retention occurs in the MYCN + /SNRPD3- sample whereas our in silico RNA-seq data shows that other samples incorporate exon 3 in BIRC5 or splice out intron 7 in CDK10 in the final mRNA transcript (Fig. 4E). The RNA-seq findings were validated in vitro by RT-qPCR using two sets of primers: one that recognised preceding exons in BIRC5 or CDK10, and, a second primer pair recognising the BIRC5 skipped exon or the CDK10 retained intron (Supplementary Fig. 3D). RT-qPCR analysis using both primer pairs revealed non-significant changes to total *BIRC5* expression but a significant reduction in exon 3 expression in MYCN + /SNRPD3- samples compared to MYCN-/SNRPD3+ (wildtype) samples, for CDK10 there was a significant reduction in total CDK10 expression and a significant increase in intron 7 retention (Supplementary Fig. 3E). Considering both expression changes in the skipped exon or retained intron and total mRNA, we confirmed significant skipping of exon 3 in *BIRC5* and intron 7 retention in *CDK10* resulting from SNRPD3 knockdown, and therefore, favouring the expression of the alternative isoform (labelled Isoform b) (Supplementary Fig. 3F). Taken together with our finding that knockdown of SNRPD3 in MYCN-high expressing cells resulted in greater reduction of cell viability when compared to MYCN-low expressing cells (Fig. 2G), we propose that the skipping of exon 3 in BIRC5 and intron 7 retention in CDK10 are toxic splice events in MYCN-driven neuroblastoma which are prevented by SNRPD3 in MYCN driven cancer cells. Therefore, SNRPD3 preserves balanced MYCN-driven splicing of the cell cycle genes *BIRC5* and *CDK10*.

### MYCN, SNRPD3 and PRMT5 form a protein complex resulting in enhanced methylation of SNRPD3

**Fig. 5:**
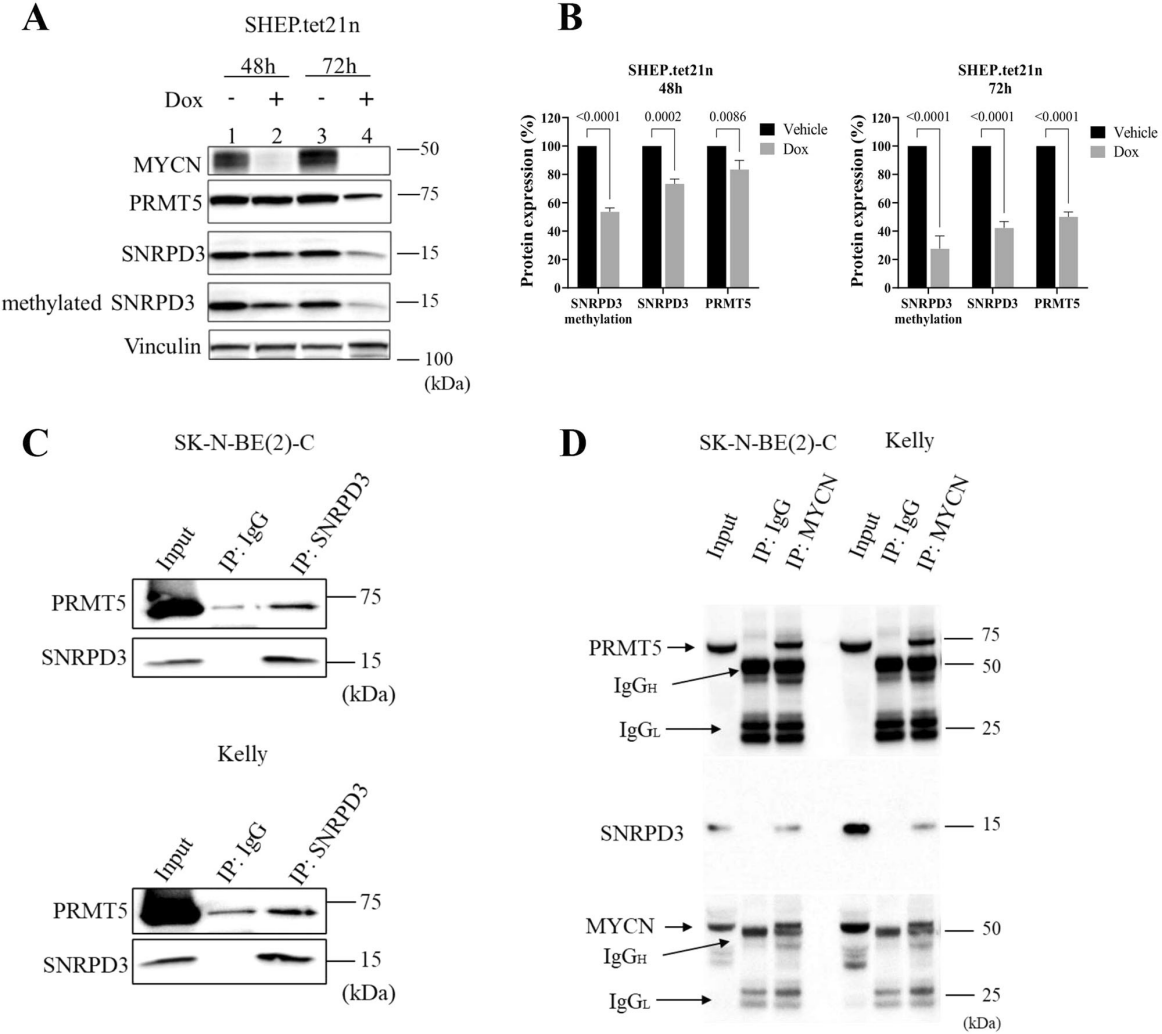
MYCN, SNRPD3 and PRMT5 form a protein complex to enhance SNRPD3 methylation. **A** Representative western blot of MYCN, PRMT5, SNRPD3 and SNRPD3 protein arginine methylation (methylated SNRPD3) expression in SHEP.tet21n cells following treatment with either vehicle or Dox at 48 and 72 h. **B** Densitometry analysis of SNRPD3 protein arginine methylation, PRMT5 and SNRPD3 protein expression in SHEP.tet21n cells following treatment with either vehicle or Dox at 48 and 72 h. **C** Representative western blot for endogenous PRMT5 and SNRPD3 after immunoprecipitation of SNRPD3 from SK-N-BE(2)-C and KELLY cells. Five percent of the cell lysate was loaded as input. **D** Representative western blot for endogenous SNRPD3, PRMT5 and MYCN after immunoprecipitation of MYCN from SK-N-BE(2)-C or KELLY cells. Five percent of the cell lysate was loaded as input. All results represent *n* = 3 independent biological replicates, mean ± SEM, where the *P* value was determined through a two-way ANOVA, with Tukey’s multiple comparison tests.

As previously shown, core spliceosome genes are globally upregulated in the presence of MYCN amplification, one of which is *SNRPD3*. We examined SNRPD3 methylation in response to changing MYCN expression. The western blot analysis in SHEP.tet21n cells demonstrated a reduction in SNRPD3 methylation in response to Dox treatment (Fig. 5A, B). The reduction in methylation was shown to be time-dependent, where the greatest reduction is observed at 72 h (lane 2 vs lane 4, Fig. 5A, B). The reduction of SNRPD3 methylation observed at 48 h was greater than the reduction in total SNRPD3 and PRMT5 protein expression (lane 1 vs lane 2, Fig. 5A, B), indicating that the reduction in SNRPD3 methylation was not due entirely to reduced SNRPD3 levels. These results suggest that MYCN expression influences the levels of SNRPD3 methylation.

We next investigated whether SNRPD3, MYCN, and PRMT5 proteins directly bound each other. Since the methylation of SNRPD3 is an important step in its function and is catalysed by PRMT5, an SNRPD3 co-immunoprecipitation (Co-IP) was performed. Endogenous SNRPD3 was immunoprecipitated from *MYCN*-amplified neuroblastoma, SK-N-BE(2)-C and KELLY cell lines and PRMT5 protein was shown to bind SNRPD3 (Fig. 5C). We hypothesised that MYCN might also form a protein complex with SNRPD3 and/or PRMT5 [34] to influence the methylation of SNRPD3 and thus the function of the spliceosome. A MYCN Co-IP demonstrated that endogenous MYCN bound both SNRPD3 and PRMT5 in both SK-N-BE(2)-C (lane 1–3) and KELLY (lane 4–6) cell lines (Fig. 5D). Together these findings suggest that MYCN, SNRPD3, and PRMT5 proteins may cooperatively complex to enhance SNRPD3 methylation and spliceosome assembly in MYCN-driven neuroblastoma.

### Chemical inhibition of PRMT5 reduces the survival and proliferation of neuroblastoma cells in a MYCN and SNRPD3-dependent manner

**Fig. 6:**
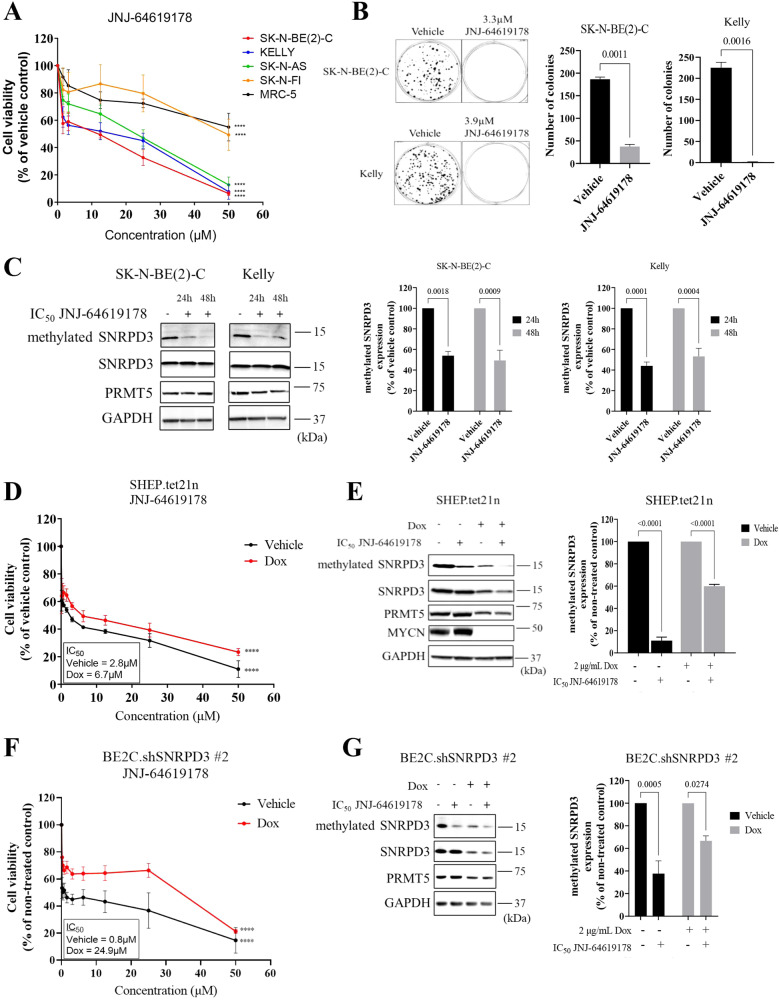
Chemical inhibition of PRMT5 has selective toxicity for neuroblastoma cells compared to normal myofibroblast cells. **A** Cell viability measured 72 h after SK-N-BE(2)-C, KELLY, SH-SY5Y, SK-N-FI, and MRC-5 cells were treated with increasing concentrations (0–50 µM) of the PRMT5 inhibitor, JNJ-64619178. **B** SK-N-BE(2)-C and KELLY cells were treated with IC_50_ concentrations of JNJ-64619178 for 10 days (SK-N-BE(2)-C) or 14 days (KELLY), followed by colony formation assays. Quantification of colony forming assays was based on colony numbers. Differences in colony formation were compared to the vehicle control. All results represent *n* = 3 independent biological replicates, mean ± SEM, where the *P* value was determined through a one-way ANOVA, with Dunnett multiple comparison testing. **C** Representative western blot of SNRPD3 protein arginine methylation (methylated SNRPD3), SNRPD3, and PRMT5 expression following treatment with IC_50_ concentration of JNJ-64619178 in SK-N-BE(2)-C and KELLY cells for 24 and 48 h, followed by densitometry analysis, where difference in protein expression was compared to vehicle control. **D** Cell viability was measured 72 h after vehicle or Dox treated SHEP.tet21n cells were treated with increasing concentrations (0–50 µM) of JNJ-64619178. **E** Representative western blot of SNRPD3 protein arginine methylation, SNRPD3, PRMT5, and MYCN expression following treatment of vehicle or Dox treated SHEP.tet21n cells with IC_50_ concentrations of JNJ-64619178 for 24 h, followed by densitometric analysis of protein expression compared to vehicle control. **F** Cell viability was measured 72 h after vehicle or Dox treated BE(2)-C.shSNRPD3 #2 cells were treated with increasing concentrations (0–50 µM) of JNJ-64619178. **G** Representative western blot of SNRPD3 protein arginine methylation, SNRPD3, and PRMT5 expression following treatment of vehicle or Dox treated BE2C.shSNRPD3 #2 cells with IC_5__0_ concentrations of JNJ-64619178 for 24 h, followed by densitometry analysis of protein expression compared to vehicle control. Two-way ANOVA statistical test was performed for each concentration compared back to no drug control (at 0 μM) (****p < 0.0001). All results represent *n* = 3 independent biological replicates, mean ± SEM, where the *P* value was determined through a two-way ANOVA, with Tukey’s multiple comparison tests, unless stated otherwise.

To investigate whether targeting SNRPD3 methylation is an effective therapeutic strategy in *MYCN*-amplified neuroblastoma we assessed the effects of the PRMT5 inhibitor, JNJ-64619178 in vitro. JNJ-64619178 simultaneously binds the S-adenosyl-L-methionine (SAM) and protein substrate binding pockets of the PRMT5/MEP50 complex to prevent SNRPD3 methylation [34]. JNJ-64619178 was screened at a range of concentrations (0–50 µM) for its effects on cell viability on *MYCN*-amplified (SK-N-BE(2)-C and KELLY), *MYCN* non-amplified (SH-SY5Y and SK-N-FI) neuroblastoma cell lines, and the normal lung fibroblast, MRC-5. JNJ-64619178 potently reduced cell viability in both *MYCN*-amplified and non-amplified neuroblastoma cell lines, apart from SK-N-F1, compared to the normal fibroblast cell line (Fig. 6A and Supplementary Table 3). Interestingly, the SK-N-FI cell line has little to no MYCN and MYC expression, whereas the SH-SY5Y cell line has high MYC expression. Moreover, SK-N-BE(2)-C and KELLY cells treated with the IC_50_ concentrations of JNJ-64619178 exhibited a near complete ablation of colony forming capacity (Fig. 6B). To determine if JNJ-64619178 was targeting SNRPD3 methylation, we assessed protein expression and observed that treatment significantly reduced methylated SNRPD3 protein levels with minimal reduction in SNRPD3 and PRMT5 overall protein levels in both SK-N-BE(2)-C and KELLY cells, with reductions observed as early as 24 h (Fig. 6C and Supplementary Fig. 4A).

We then evaluated whether JNJ-64619178 exhibited MYCN dependency in its effects on neuroblastoma cell viability. SHEP.tet21n cells were first treated with Dox to reduce MYCN, followed by JNJ-64619178 at a range of concentrations (0–50 µM). Cell viability was then measured after 72 h. We found that JNJ-64619178 was more potent in the presence of MYCN (IC_50_ of 2.8 μM), as measured by IC_50_, compared to Dox-treated cells (IC_50_ of 6.7 μM) (Fig. 6D). Western blot analysis confirmed successful knockdown of MYCN, along with a reduction in SNRPD3 methylation (Fig. 6E). As expected, a greater reduction of SNRPD3 protein expression was observed with the MYCN knockdown (Supplementary Fig. 4B).

As PRMT5 inhibitors affect other protein methylation events, we assessed whether JNJ-64619178 effects on neuroblastoma cell viability were dependent on SNRPD3 in neuroblastoma cells. Dox-inducible, SNRPD3 or control shRNA-expressing SK-N-BE(2)-C cells were treated at a range of concentrations (0–50 µM) and cell viability was measured. KELLY cells were transfected with either SNRPD3 or control siRNAs for 72 h, followed by a secondary transfection with control or SNRPD3 siRNA for 24 h before treatment with increasing concentrations (0–50 µM) of JNJ-64619178. We found that JNJ-64619178 was more potent in the presence of SNRPD3 (IC_50_ of 0.8 μM for BE2C.shSNRPD3 #2 (vehicle); IC_50_ of 2.3 μM for BE2C.shSNRPD3 #1(vehicle); IC_50_ of 0.5 μM for KELLY siControl), compared to the Dox-treated or SNRPD3 siRNA transfected cells (IC_50_ of 24.9 μΜ for BE2C.shSNRPD3 #2; IC_50_ of 60.5 μM for BE2C.shSNRPD3 #1; IC_50_ of 12.0 μM for KELLY siSNRPD3 #1; IC_50_ of 5.4 μM for KELLY siSNRPD3 #2) (Fig. 6F and Supplementary Fig. 4C, D). We found no difference in cell viability between vehicle and Dox-treated BE2C.shControl cells (Supplementary Fig. 4E). Western blot analysis confirmed the successful knockdown of SNRPD3, along with a reduction in SNRPD3 methylation after treatment with JNJ-64619178 in both BE2C.shSNRPD3 #2 (Fig. 6G and Supplementary Fig. 4F) and BE2C.shSNRPD3 #1 (Supplementary Fig. 4G, H), and KELLY cells transfected with SNRPD3 siRNA (Supplementary Fig. 4I, J). Lastly, western blot analysis of protein from BE2C.shControl cell lines showed a reduction of methylated SNRPD3 but no significant reduction in total SNRPD3 or PRMT5 after treatment with JNJ-64619178 (Supplementary Fig. 4K, L). These results reveal a novel therapeutic strategy for targeting SNRPD3 methylation in *MYCN*-amplified neuroblastoma.

## Discussion

Here we have determined that a core spliceosome protein, SNRPD3, plays a vital role in maintaining the fidelity and balance of MYCN-driven alternative splicing events which are required to promote neuroblastoma tumorigenesis. Therapeutic inhibition of SNRPD3 methylation and thus spliceosome function using a PRMT5 inhibitor was an effective strategy for reducing cell viability in a SNRPD3- and MYCN-dependent manner. Our findings extend our understanding of global gene dysregulation caused by the MYCN oncoprotein, point to specific alternative splicing events required for MYCN oncogenesis, and suggests a potential role for therapeutics which block SNRPD3 methylation and its binding to MYCN.

Alternative splicing is a tightly regulated process to ensure a delicate balance between isoforms is maintained for normal cell functioning. In cancer cells this balance is no longer maintained, and alternative mRNA isoforms that promote tumorigenesis are expressed [35]. By altering the splicing machinery to gain growth advantage, cancer cells may be sustaining a trade-off between splicing efficiency and splicing fidelity [36]. Our data suggests that cancer-related alternative splicing can be easily disrupted by placing excess demand on the splicing machinery through depletion of SNRPD3, thereby reducing cell viability. The methylation of Sm proteins through PRMT5 has been shown to ensure splicing fidelity in MYC-driven lymphomas, where the expression of these proteins are up-regulated [28]. We hypothesise that a similar mechanism is utilised in neuroblastoma to maintain splicing fidelity, where MYCN increases the expression of PRMT5, SNRPD3, and SNRPD3 methylation. Additionally, we show for the first time that MYCN directly interacts with the splicing machinery by binding to SNRPD3 and PRMT5. PRMT5 has been reported to bind MYCN resulting in the methylation of arginine residues in MYCN [37]. The exact functional outcome of the MYCN, SNRPD3, and PRMT5 protein complex requires further investigation, however, our findings suggest that MYCN may facilitate the binding between PRMT5 and SNRPD3 to increase SNRPD3 protein arginine methylation. Moreover, our study along with others are beginning to reveal that proto-oncogenes such as MYC and MYCN can regulate the methylation of splicing factors that provide cancer cells with a mechanism which promotes cell survival [28, 38]. Furthermore, one limitation from bulk RNA analyses of this study is that they don’t account for cellularity and therefore transcriptional activity of each cell, which may relate to the expression of spliceosome genes and splicing activity. Newer single-cell analyses exploring alternate splicing patterns when SNRPD3 or MYCN are perturbed will be valuable to address this limitation.

The splicing specificity created by SNRPD3 and MYCN alongside the observed 100% survival in xenografted mice with depleted SNRPD3 expression suggests that neuroblastoma cancer cells are susceptible to therapeutic inhibition of SNRPD3. Indeed, the current study showed that the PRMT5 inhibitor, JNJ-64619178, is effective against neuroblastoma cell lines and demonstrated SNRPD3- and MYCN-dependency. PRMT5 inhibitor-sensitive cells may have an addiction to excess alternative splicing that leads to a therapeutic vulnerability [39, 40]. However, we do note that the observed MYCN-dependency may reflect the initial chemosensitivity that is exhibited in *MYCN*-amplified neuroblastomas. Therefore, further exploration of combining PRMT5 and chemotherapeutics is warranted. This is especially relevant as recent studies have suggested that PRMT5 inhibitors can increase the sensitivity of cancer cells to chemotherapeutics, even in resistant populations [41, 42]. Consequently SNRPD3, MYCN, and PRMT5 expression levels may be used as predictive biomarkers for PRMT5 inhibitor response, not only in neuroblastoma, but potentially other cancers.

Our data adds to the growing body of evidence indicating cell cycle pathways are profoundly affected by SNRPD3 or PRMT5 inhibition [20, 21, 38, 39, 43, 44], suggesting that combination therapy between PRMT5 and cell cycle inhibitors may be a promising avenue for further exploration in cancer therapeutics. We revealed that BIRC5 (*Survivin*) and CDK10 were the most differentially spliced cell cycle genes resulting from SNRPD3 depletion, when MYCN is overexpressed. BIRC5 is a well-established contributor to cancer progression in several cancers [45–48], including neuroblastoma [49–51]. However, BIRC5 targeted agents have failed to progress to the clinic [48]. One of the greatest challenges faced by BIRC5 inhibitors in clinical trials is low antitumour efficacy and high toxicity [48, 52, 53]. Thus, combination treatment with PRMT5 and BIRC5 inhibitors has the potential to increase the therapeutic window and achieve greater efficacy.

Overall, this study has provided novel insights into the molecular mechanisms of SNRPD3 as an oncogenic protein, and alternative splicing in MYCN-driven neuroblastoma. We show that in neuroblastoma there is both, a reliance on a hyperactivated spliceosome, and a fidelity which is maintained by SNRPD3. Our findings highlight that disruption of SNRPD3 protein methylation using PRMT5 inhibitors is a promising novel therapeutic target for the treatment of high-risk neuroblastoma, particularly given the observed SNRPD3- and MYCN-selectivity.

## Materials and methods

### Cell culture

Neuroblastoma cell lines, SK-N-BE(2)-C, SK-N-AS, SH-SY5Y, SK-N-FI, IMR-32, and SHEP.tet21N were maintained in Dulbecco’s modified Eagle’s medium (DMEM) (Life technologies Australia, VIC, Australia) with 10% foetal calf serum (FCS) (Life Technologies). CHP-134 and KELLY cells were cultured in RPMI media (Life Technologies) with 10% FCS. MRC-5 and WI-38 normal human fibroblasts were grown in alpha-minimum essential media (MEM) (Life Technologies) supplemented with 10% FCS. Neuroblastoma cell line SHEP.tet21N, which are genetically modified to repress human MYCN cDNA when exposed to doxycycline (dox) was kindly provided by Professor Jason Shohet (Texas Children’s Cancer Center, Houston, TX, USA). Neuroblastoma cell lines, SK-N-BE(2)-C, and SH-SY5Y cells were provided by Barbara Spengler (Fordham University, New York, NY). The neuroblastoma cell line, IMR-32, and SK-N-FI, along with the normal human fibroblasts, MRC-5 and WI-38 cells were obtained from the American Type Culture Collection (ATCC) (Manassas, VA, USA). Neuroblastoma cell lines, KELLY, CHP-134, and SK-N-AS cells were obtained from the European Collection of Cell Cultures through Sigma-Aldrich (Sigma-Aldrich, Sydney, Australia). All cell lines used were authenticated by Cell Bank Australia (Westmead, NSW, Australia), STR validated and free from mycoplasma, and cultured at 37 °C/5% CO_2_ in a humidifier incubator.

### Establishment of doxycycline-inducible control shRNA and SNRPD3 shRNA-expressing neuroblastoma cell lines

To generate control and stable SNRPD3-inducible knockdown cells, SK-N-BE(2)-C cells were transduced with pSMART non-targeting control vector or pSMART encoding a SNRPD3-specific shRNA’s (shSNRPD3#1: 5’-TTAACATGGGTGCGTTCTT-3’ or shSNRPD3 #2: 5’-CCCGATATACCTCACCGGT-3’) along with 8 μg/ml of polybrene (Santa Cruz Biotechnology). SK-N-BE(2)-C cells were then incubated with 2 μg/ml of puromycin (Sigma-Aldrich) to select for positive clones.

### In vivo mouse experiments

All experimental procedures involving mice were approved by the University of New South Wales Animal Care and Ethics Committee (ACEC# 21/29B) according to the Animal Research Act, 1985 (New South Wales, Australia) and the Australian Code of Practice for Care and Use of Animals for Scientific Purposes (2013). Subcutaneous xenograft models of neuroblastoma were established by subcutaneous injection of 2 × 10^6^ doxycycline-inducible SNRPD3 shRNA SK-N-BE(2)-C cells into the right flank of 5–6 week old female Balb/c nude mice. When engrafted tumours reached 4−5 mm, mice were randomised into 2 groups: Vehicle (5% Sucrose) or doxycycline (Dox)-treated mice. Mice with undetectable engraftment were excluded. Mice were fed with 5% sucrose water with or without doxycycline hyclate (Sigma) at 2 mg/mL. Tumour development was monitored, and tumour volume was calculated using 0.5 × (length × width × height). Mice were culled when tumour volume reached 1000 mm^3^ or reached the maximum holding time of 12 weeks. Investigators were not blinded to the treatment given.

### Microarray analysis on Th-*MYCN*^+/+^ ganglia tissues

Ganglia from male and female Th-*MYCN*^+/+^ mice and wild-type controls were collected at 2, 4, and 6 weeks of age (on 129 × 1/SvJ strain). RNA was extracted from the cells with a PureLink RNA Kit (Life Technologies), and genome-wide differential gene expression was examined using Agilent SurePrint G3 Mouse GE 8 × 60 K Microarrays. The microarray data was analyzed in R [http://www.r-project.org] and normalised using the vsn method using the “limma” package [29, 54].

### Human neuroblastoma tumour gene expression and survival analysis

Microarray data were obtained for 649 clinically annotated primary neuroblastoma samples (KOCAK neuroblastoma cohort) from the gene mRNA expression omnibus (GEO) with the accession GSE45547 [55]. Orthogonal analyses were conducted in an independent gene expression microarray dataset, consisting of 498 clinically annotated primary neuroblastoma samples (SEQC neuroblastoma cohort) from GEO with the accession GSE49711 [56]. The *survival* R package was used to run univariate or multivariate Cox Proportional Hazards (CoxPH) regression models for genes of interest using median gene expression. Followed by regressing covariates associated with neuroblastoma prognosis such as advanced disease stage (3 and 4), age at diagnosis (>18 months), and MYCN-amplification status. We utilised matched clinical annotations containing event-free and overall survival data to construct Kaplan–Meier survival curves of subgroups dichotomised by median gene expression using the *survival* R package. All statistical tests concerning Kaplan–Meier analyses were done using log-rank tests, adjusted using the Bonferroni method for multiple hypotheses testing where appropriate. Correlation of gene expression between genes of interest as well as with gene copy number, was analysed using Pearson correlation and resultant *p*-values were adjusted using the Bonferroni method.

### siRNA transfections

For siRNA mediated knockdown, the indicated cell lines were transfected with 20 nM of siGENOME plus human SNRPD3 siRNA (SNRPD3 siRNA #1: 5’- GAAGAACGCACCCATGTTA-3’ and SNRPD3 siRNA #2: 5’- GAACACCGGTGAGGTATAT-3’) synthesised by Dharmacon. Non-targeting pool siRNA was used as a siControl and purchased from Dharmacon. Cells were transfected for 24 h to 72 h after plating, depending on the experimental requirements. A secondary transfection was performed in KELLY cells 72 h post first transfection, for a total of 96 h of knockdown before JNJ-64619178 treatment. Transfections were performed using Lipofectamine 2000 (Life technologies) following the manufacturer’s instructions.

### RNA isolation and quantitative real-time PCR

Total RNA was extracted using the PureLink RNA kit (Life Technologies) according to the manufacturer’s protocol. cDNA was synthesised by reverse-transcribing 1 μg of RNA using the Tetro cDNA synthesis kit (Bioline) according to manufacturer’s protocol. To quantify gene expression using RT-qPCR, 2 μL of cDNA was added to a reaction mix containing: 10 μL 10x Power SYBR Green mix (Applied Biosystems, Life Technologies), 1 μL Forward primer, 1 μL Reverse primer, and 6uL of DEPC-treated H_2_O. Differential gene expression was measured on the Quantstudio^TM^ 3 (ThermoFisher Scientific) using the standard protocol and data analysed using the log2ΔΔCt method. All mRNA expression levels were normalised to glyceraldehyde-3-phosphate dehydrogenase (GAPDH). Primer sequences (Supplementary Table 1) were obtained from Integrated DNA Technologies (NSW, Australia).

### RNA-sequencing and analysis

RNA-seq libraries were prepared using TruSeq Stranded mRNA-seq (Illumina); paired-end sequencing (NovaSeq 6000, 2 × 100 base pairs) was performed by Ramaciotti Centre of Genomics (UNSW, Sydney, Australia). Reads were mapped to hg38 human genome assembly using STAR (v2.5.0a) with the recommended ENCODE parameters and appropriate adjustments [57]. Differential splicing was conducted using the leafcutter alternative splicing tool. Aligned reads were passed through the leafcutter workflow and differential results obtained [32]. Annotations through leafcutter were made using the GENCODE v28 annotation. Statistically significant differential splicing events were determined by leafcutter across all pairwise sample comparisons (false discovery rate <0.05). Delta PSI was computed using the average value of the MYCN-/SNRPD3+ condition as a reference. Adjusted delta-PSI value represents all delta PSI values in terms of absolute value, except when delta-PSI for a particular sample differed to the more prevalent direction of delta PSI across all samples. These values are represented in the form of a heatmap (ComplexHeatmap package) [58]. To determine differential splicing events, rMATS (v4.1.0) [33] was used to count junction reads and reads falling into the tested region within ENSEMBL gene definitions. Splicing events were labelled significant if FDR < 0.001 and Inclusion Level Difference (ΔΨ) > │−1.5│.

### Immunoprecipitation

For endogenous MYCN and SNRPD3 immunoprecipitation assays, SK-N-BE(2)-C and KELLY cells were lysed in cold BC100 (Sigma-Aldrich) or NP40 buffer (Sigma-Aldrich), respectively, supplemented with protease inhibitor (Sigma-Aldrich). Five percent of the cell extract was saved as the input, the remainder (750 μg of total protein) was incubated with either 10 μg MYCN-specific antibody (Merck Millipore Millipore, VIC, Australia) or 5 μg SNRPD3-specific antibody (Abcam, ab157118) or control IgG antibody (10 μg for mouse (Santa Cruz Biotechnology) or 5 μg for rabbit (Cell signalling)) and A/G PLUS agarose beads (Bio-Strategy) at 4 °C overnight. After 3 washes with the lysis buffer, the bound proteins were eluted by boiling with SDS sample buffer. Bound proteins were resolved by SDS-PAGE.

### Western blot analysis

Cell pellets were lysed in RIPA buffer (Sigma-Aldrich) supplemented with protease inhibitor (Sigma-Aldrich) and incubated on ice for 30 min. After centrifugation at 12,000 x g for 20 min at 4 °C, the supernatant was collected. Protein concentration was determined using the BCA protein quantification assay kit as per manufacturer’s instructions (ThermoFisher Scientific, Waltham, MA, USA), and 15–30 μg whole protein lysates were resolved on either the 10–20% Criterion™ Tris-HCl Protein Gel (Bio-rad) or the NuPAGE™ 4 to 12%, Bis-Tris mini protein gel (Invitrogen). Nitrocellulose membranes (GE Healthcare) were blocked with 10% (wt/vol) nonfat dry milk in Tris-buffered saline with Tween-20 (20 mM Tris-HCl (pH 7.6), 137 mM NaCl, 0.1% Tween-20), then incubated overnight at 4 °C with the following primary antibodies: SNRPD3 (1:500; HPA001170; Sigma-Aldrich), MYCN (1:1000; B84B; Santa Cruz Biotechnologies), PRMT5 (1:1000; sc-376937; Santa Cruz Biotechnology), SYM11 (1:500; 07-413; Merck Millipore), GAPDH (1:2000; G-9; Santa Cruz Biotechnology), cMYC (1:500; D84C12; Cell Signalling Technologies), and Vinculin (1:10000; V9131; Sigma-Aldrich). Appropriate horseradish peroxidase-conjugated secondary antibodies (1:1000; Santa Cruz Biotechnologies and Merck Millipore) were diluted in 1% (wt/vol) nonfat dry milk in Tris-buffered saline with Tween-20 and membranes were probed at room temperature for 2 h. Immunoblots were visualised with Clarity ECL Chemiluminescence reagents (Bio-Rad). Densitometry of protein expression was measured using Image Lab software (BioRad) and each protein expression band was normalised to loading controls.

### Chromatin immunoprecipitation assay

Preparation of DNA from SK-N-BE(2)-C and KELLY cells for ChIP assay were performed using the Chromatin-Immunoprecipitation Assay kit (Merck Millipore) according to the manufacturer’s instructions. ChIP assays were performed with mouse anti-MYCN antibody (B84B; Santa Cruz Biotechnology) or control mouse IgG antibody (Santa Cruz Biotechnology). DNA was purified using a MiniElute PCR Purification Kit (Qiagen). Real-time PCR was performed with primers designed targeting a negative control region (−2000 bp downstream of *SNRPD3* TSS) or SNRPD3 target sequence (+647 bp upstream from SNRPD3 TSS). Fold enrichment of SNRPD3 target region was calculated by dividing cycle threshold values of the SNRPD3 target region by cycle threshold values of the negative control region, relative to input. Primer sequences (Supplementary Table 1) were obtained from Integrated DNA Technologies (NSW, Australia).

### Drug treatments

Neuroblastoma cell lines or normal human fibroblasts were seeded in either a 96-well plate, 6-well plate or T75 flask (Corning) at densities that would reach 70–90% confluency at the 24–72 h assay endpoint. All inducible cell lines were primed with 2 μg/ml of Dox 72 h prior to drug treatments. Following an initial 24 h incubation after seeding, drug treatments were made at the specified concentrations in fresh media. JNJ-64619178 (HY-101564; MedChemExpress) was constituted in DMSO (Sigma-Aldrich). Cells were incubated at 37 °C with 5% CO2 for 24–96 h, followed by various phenotypic assays.

### Cell proliferation assay

Cell proliferation was measured using the Cell Proliferation ELISA, BrdU colorimetric assay (11647229001, Roche) according to the manufacturer’s instructions. Changes in cell proliferation were calculated from the absorbance readings at 370 nm (490 nm reference wavelength) on the Benchmark Plus microplate reader (Bio-Rad).

### Colony forming assay

A total of 500 SK-N-BE(2)-C and 500 KELLY cells, transfected with either control siRNA or SNRPD3 siRNA #1 or SNRPD3 siRNA #2, were seeded in 6-well plates and left to grow for 10 and 14 days, respectively. A total of 500 SK-N-BE(2)-C cells expressing shSNRPD3 or shControl constructs or 200 SHEP.tet21n cells were seeded in 6-well plates and treated with or without Dox for 72 h, then left to grow for 10 days to allow colonies to form. Five hundred SK-N-BE(2)-C and KELLY cells were seeded in 6-well plates and treated with JNJ-64619178 for 72 h then left to grow for 10 and 14 days, respectively. Colonies were fixed and stained in crystal violet containing 50% methanol. Stained colonies were then imaged and quantified using Image J.

### Cell viability assay

Cell viability was measured using the Alamar Blue fluorescence assay (Invitrogen) according to the manufacturer’s instructions. Assay absorbance was determined using the Wallac 1420 VICTOR2^TM^ microplate reader at an excitation/emission of 560/590 nm (PerkinElmer). A baseline reading was taken at 0 h, followed by readings after 6 h. All data were normalised and compared to a treatment control.

### Flow cytometry

Cell cycle phases and apoptosis among treated cells were estimated using Propidium Iodide (556463, BD Pharmingen) staining. SK-N-BE(2)-C and KELLY adherent and floating cells were harvested and adjusted to an equal number of cells per sample. Cells were fixed in ethanol overnight then stained with Propidium Iodide. Samples were analysed by flow cytometry using the Fortessa (BD Biosciences, Macquarie Park, NSW, AU) and the data was analysed using the FlowJo software (Ashland, OR, USA).

### Statistical analysis

All experiments conducted were performed a minimum of three independent experiments (*n* = 3) unless stated otherwise. For animal studies, seven mice (*n* = 7) were used per treatment group. Data obtained from all experiments were analysed using the software GraphPad prism 9. Statistical analysis to determine *P* values was completed using either; one-way or two-way ANOVA, with Tukey’s multiple comparison tests, or unpaired *t* tests. All values are expressed as mean values with error bars representing ± SEM. Power calculations for estimation of sample size were not used for all in vitro and in vivo experiments and sample size was chosen based on prior experience in similar studies. Samples were not excluded from analyses unless deemed to have technical errors or problems.

### Supplementary information

Supplementary Figures

## Data Availability

The data generated in this study are available within the article and its supplementary data files. The RNA-seq data generated in this study were deposited in the Gene Expression Omnibus (GEO) and is publicly available under the GEO accession number [GSE213762]. The Th-MYCN ganglia microarray data analysed in this study was obtained from the ArrayExpress database with the accession number E-MTAB-3247. The Kocak and SEQC neuroblastoma patient tumour cohort data analysed in this study were obtained from the Gene Expression Omnibus (GEO) with the accession GSE45547 and GSE62564.

## Code availability

Code for bioinformatics analyses produced in R can be obtained by request to the corresponding author.

## References

[1] Pinto NR, Applebaum MA, Volchenboum SL, Matthay KK, London WB, Ambros PF (2015). Advances in risk classification and treatment strategies for neuroblastoma. J Clin Oncol.

[2] Gustafson WC, Meyerowitz JG, Nekritz EA, Chen J, Benes C, Charron E (2014). Drugging MYCN through an allosteric transition in Aurora kinase A. Cancer cell.

[3] Zeid R, Lawlor MA, Poon E, Reyes JM, Fulciniti M, Lopez MA (2018). Enhancer invasion shapes MYCN-dependent transcriptional amplification in neuroblastoma. Nat Genet.

[4] Eilers M, Eisenman RN (2008). Myc’s broad reach. Genes Dev.

[5] Huang M, Weiss WA (2013). Neuroblastoma and MYCN. Cold Spring Harb Perspect Med.

[6] David CJ, Chen M, Assanah M, Canoll P, Manley JL (2010). HnRNP proteins controlled by c-Myc deregulate pyruvate kinase mRNA splicing in cancer. Nature.

[7] Anczuków O, Akerman M, Clery A, Wu J, Shen C, Shirole NH (2015). SRSF1-regulated alternative splicing in breast cancer. Mol cell.

[8] Ladomery M. Aberrant alternative splicing is another hallmark of cancer. Int J Cell Biol. 2013;2013:463786. 10.1155/2013/463786 PMC3786539 24101931

[9] Zhou Y, Han C, Wang E, Lorch AH, Serafin V, Cho B-K (2020). Posttranslational regulation of the exon skipping machinery controls aberrant splicing in leukemia. Cancer Discov.

[10] El Marabti E, Younis I (2018). The cancer spliceome: reprograming of alternative splicing in cancer. Front Mol Biosci.

[11] Jurica MS, Moore MJ (2003). Pre-mRNA splicing: awash in a sea of proteins. Mol cell.

[12] Palacios I, Hetzer M, Adam SA, Mattaj IW (1997). Nuclear import of U snRNPs requires importin β. EMBO J.

[13] Urlaub H, Raker VA, Kostka S, Lührmann R (2001). Sm protein–Sm site RNA interactions within the inner ring of the spliceosomal snRNP core structure. EMBO J.

[14] Meister G, Eggert C, Bühler D, Brahms H, Kambach C, Fischer U (2001). Methylation of Sm proteins by a complex containing PRMT5 and the putative U snRNP assembly factor pICln. Curr Biol.

[15] Friesen WJ, Paushkin S, Wyce A, Massenet S, Pesiridis GS, Van Duyne G (2001). The methylosome, a 20S complex containing JBP1 and pICln, produces dimethylarginine-modified Sm proteins. Mol Cell Biol.

[16] Blijlevens M, van der Meulen-Muileman IH, de Menezes RX, Smit EF, van Beusechem VW (2019). High-throughput RNAi screening reveals cancer-selective lethal targets in the RNA spliceosome. Oncogene.

[17] Cunha IW, Carvalho KC, Martins WK, Marques SM, Muto NH, Falzoni R (2010). Identification of genes associated with local aggressiveness and metastatic behavior in soft tissue tumors. Transl Oncol.

[18] Blijlevens M, Komor MA, Sciarrillo R, Smit EF, Fijneman RJ, van Beusechem VW (2020). Silencing core spliceosome Sm gene expression induces a cytotoxic splicing switch in the proteasome subunit beta 3 mRNA in non-small cell lung cancer cells. Int J Mol Sci.

[19] Koedoot E, van Steijn E, Vermeer M, González-Prieto R, Vertegaal AC, Martens JW (2021). Splicing factors control triple-negative breast cancer cell mitosis through SUN2 interaction and sororin intron retention. J Exp Clin Cancer Res.

[20] Siebring-van Olst E, Blijlevens M, de Menezes RX, van der Meulen-Muileman IH, Smit EF, van Beusechem VW (2017). A genome-wide si RNA screen for regulators of tumor suppressor p53 activity in human non-small cell lung cancer cells identifies components of the RNA splicing machinery as targets for anticancer treatment. Mol Oncol.

[21] Weng M-T, Lee J-H, Wei S-C, Li Q, Shahamatdar S, Hsu D (2012). Evolutionarily conserved protein ERH controls CENP-E mRNA splicing and is required for the survival of KRAS mutant cancer cells. Proc Natl Acad Sci.

[22] Tacconelli A, Farina AR, Cappabianca L, DeSantis G, Tessitore A, Vetuschi A (2004). TrkA alternative splicing: a regulated tumor-promoting switch in human neuroblastoma. Cancer cell.

[23] Guo X, Chen Q-R, Song YK, Wei JS, Khan J (2011). Exon array analysis reveals neuroblastoma tumors have distinct alternative splicing patterns according to stage and MYCN amplification status. BMC Med Genom.

[24] Zhang S, Wei JS, Li SQ, Badgett TC, Song YK, Agarwal S (2016). MYCN controls an alternative RNA splicing program in high-risk metastatic neuroblastoma. Cancer Lett.

[25] Szemes M, Melegh Z, Bellamy J, Park JH, Chen B, Greenhough A (2021). Transcriptomic analyses of MYCN-regulated genes in anaplastic Wilms’ tumour cell lines reveals oncogenic pathways and potential therapeutic vulnerabilities. Cancers.

[26] Hong M, He J, Li S (2019). SNW1 regulates Notch signaling in neuroblastoma through interacting with RBPJ. Biochem Biophys Res Commun.

[27] Escobar-Hoyos LF, Penson A, Kannan R, Cho H, Pan C-H, Singh RK (2020). Altered RNA splicing by mutant p53 activates oncogenic RAS signaling in pancreatic cancer. Cancer Cell.

[28] Koh CM, Bezzi M, Low DH, Ang WX, Teo SX, Gay FP (2015). MYC regulates the core pre-mRNA splicing machinery as an essential step in lymphomagenesis. Nature.

[29] Ooi CY, Carter DR, Liu B, Mayoh C, Beckers A, Lalwani A (2018). Network modeling of microRNA–mRNA interactions in neuroblastoma tumorigenesis identifies miR-204 as a direct inhibitor of MYCN. Cancer Res.

[30] Hansford LM, Thomas WD, Keating JM, Burkhart CA, Peaston AE, Norris MD (2004). Mechanisms of embryonal tumor initiation: distinct roles for MycN expression and MYCN amplification. Proc Natl Acad Sci.

[31] Hsu TY-T, Simon LM, Neill NJ, Marcotte R, Sayad A, Bland CS (2015). The spliceosome is a therapeutic vulnerability in MYC-driven cancer. Nature.

[32] Li YI, Knowles DA, Humphrey J, Barbeira AN, Dickinson SP, Im HK (2018). Annotation-free quantification of RNA splicing using LeafCutter. Nat Genet.

[33] Shen S, Park JW, Lu Z-X, Lin L, Henry MD, Wu YN (2014). rMATS: robust and flexible detection of differential alternative splicing from replicate RNA-Seq data. Proc Natl Acad Sci.

[34] Wu T, Millar H, Gaffney D, Beke L, Mannens G, Vinken P, et al. JNJ-64619178, a selective and pseudo-irreversible PRMT5 inhibitor with potent in vitro and in vivo activity, demonstrated in several lung cancer models. Cancer Res. 2018;78 (13_Supplement): 4859. 10.1158/1538-7445.AM2018-4859.

[35] Venables JP (2006). Unbalanced alternative splicing and its significance in cancer. Bioessays.

[36] Bonnal SC, López-Oreja I, Valcárcel J (2020). Roles and mechanisms of alternative splicing in cancer—implications for care. Nat Rev Clin Oncol.

[37] Park JH, Szemes M, Vieira GC, Melegh Z, Malik S, Heesom KJ (2015). Protein arginine methyltransferase 5 is a key regulator of the MYCN oncoprotein in neuroblastoma cells. Mol Oncol.

[38] Metz PJ, Ching KA, Xie T, Cuenca PD, Niessen S, Tatlock JH (2020). Symmetric arginine dimethylation is selectively required for mRNA splicing and the initiation of type I and type III interferon signaling. Cell Rep..

[39] Braun CJ, Stanciu M, Boutz PL, Patterson JC, Calligaris D, Higuchi F (2017). Coordinated splicing of regulatory detained introns within oncogenic transcripts creates an exploitable vulnerability in malignant glioma. Cancer cell.

[40] Fong JY, Pignata L, Goy P-A, Kawabata KC, Lee SC-W, Koh CM (2019). Therapeutic targeting of RNA splicing catalysis through inhibition of protein arginine methylation. Cancer cell.

[41] Mueller HS, Fowler CE, Dalin S, Moiso E, Udomlumleart T, Garg S (2021). Acquired resistance to PRMT5 inhibition induces concomitant collateral sensitivity to paclitaxel. Proc Natl Acad Sci.

[42] Wei X, Yang J, Adair SJ, Ozturk H, Kuscu C, Lee KY (2020). Targeted CRISPR screening identifies PRMT5 as synthetic lethality combinatorial target with gemcitabine in pancreatic cancer cells. Proc Natl Acad Sci.

[43] Sachamitr P, Ho JC, Ciamponi FE, Ba-Alawi W, Coutinho FJ, Guilhamon P (2021). PRMT5 inhibition disrupts splicing and stemness in glioblastoma. Nat Commun.

[44] Bezzi M, Teo SX, Muller J, Mok WC, Sahu SK, Vardy LA (2013). Regulation of constitutive and alternative splicing by PRMT5 reveals a role for Mdm4 pre-mRNA in sensing defects in the spliceosomal machinery. Genes Dev.

[45] Li F (2005). Role of survivin and its splice variants in tumorigenesis. Br J cancer.

[46] Li J, Shi J, Sang J, Yao Y, Wang X, Su L (2015). Role of survivin in the pathogenesis of papillary thyroid carcinoma. Genet Mol Res.

[47] Liu J, Du W, Fan D (2008). Survivin: the promising target in hepatocellular carcinoma gene therapy. Cancer Biol Ther.

[48] Li F, Aljahdali I, Ling X (2019). Cancer therapeutics using survivin BIRC5 as a target: what can we do after over two decades of study?. J Exp Clin Cancer Res.

[49] Hagenbuchner J, Kiechl-Kohlendorfer U, Obexer P, Ausserlechner M (2016). BIRC5/Survivin as a target for glycolysis inhibition in high-stage neuroblastoma. Oncogene.

[50] Eckerle I, Muth D, Batzler J, Henrich K-O, Lutz W, Fischer M (2009). Regulation of BIRC5 and its isoform BIRC5-2B in neuroblastoma. Cancer Lett.

[51] Lamers F, Van Der Ploeg I, Schild L, Ebus ME, Koster J, Hansen BR (2011). Knockdown of survivin (BIRC5) causes apoptosis in neuroblastoma via mitotic catastrophe. Endocr Relat Cancer.

[52] Raetz EA, Morrison D, Romanos-Sirakis E, Gaynon P, Sposto R, Bhojwani D (2014). A phase I study of EZN-3042, a novel survivin messenger ribonucleic acid (mRNA) antagonist, administered in combination with chemotherapy in children with relapsed acute lymphoblastic leukemia (ALL): a report from the therapeutic advances in childhood leukemia and lymphoma (TACL) consortium. J Pediatr Hematol/Oncol.

[53] Wiechno P, Somer BG, Mellado B, Chłosta PL, Grau JMC, Castellano D (2014). A randomised phase 2 study combining LY2181308 sodium (survivin antisense oligonucleotide) with first-line docetaxel/prednisone in patients with castration-resistant prostate cancer. Eur Urol.

[54] Ritchie ME, Phipson B, Wu D, Hu Y, Law CW, Shi W (2015). limma powers differential expression analyses for RNA-sequencing and microarray studies. Nucleic Acids Res.

[55] Kocak H, Ackermann S, Hero B, Kahlert Y, Oberthuer A, Juraeva D (2013). Hox-C9 activates the intrinsic pathway of apoptosis and is associated with spontaneous regression in neuroblastoma. Cell Death Dis.

[56] Consortium, S. (2014). A comprehensive assessment of RNA-seq accuracy, reproducibility and information content by the sequencing quality control consortium. Nat Biotechnol.

[57] Dobin A, Davis CA, Schlesinger F, Drenkow J, Zaleski C, Jha S (2013). STAR: ultrafast universal RNA-seq aligner. Bioinformatics.

[58] Gu Z, Eils R, Schlesner M (2016). Complex heatmaps reveal patterns and correlations in multidimensional genomic data. Bioinformatics.

