# Supplementary material for: MYCN and SNRPD3 cooperate to maintain a balance of alternative splicing events that drives neuroblastoma progression
Source: Oncogene. 2023 Dec 4;43(5):363–77. doi: 10.1038/s41388-023-02897-y (PMC10824661; doi:10.1038/s41388-023-02897-y)
Supplement: Supplementary file 1 — Supplementary Figures [file 41388_2023_2897_MOESM1_ESM.pdf]

Supplementary figure 1

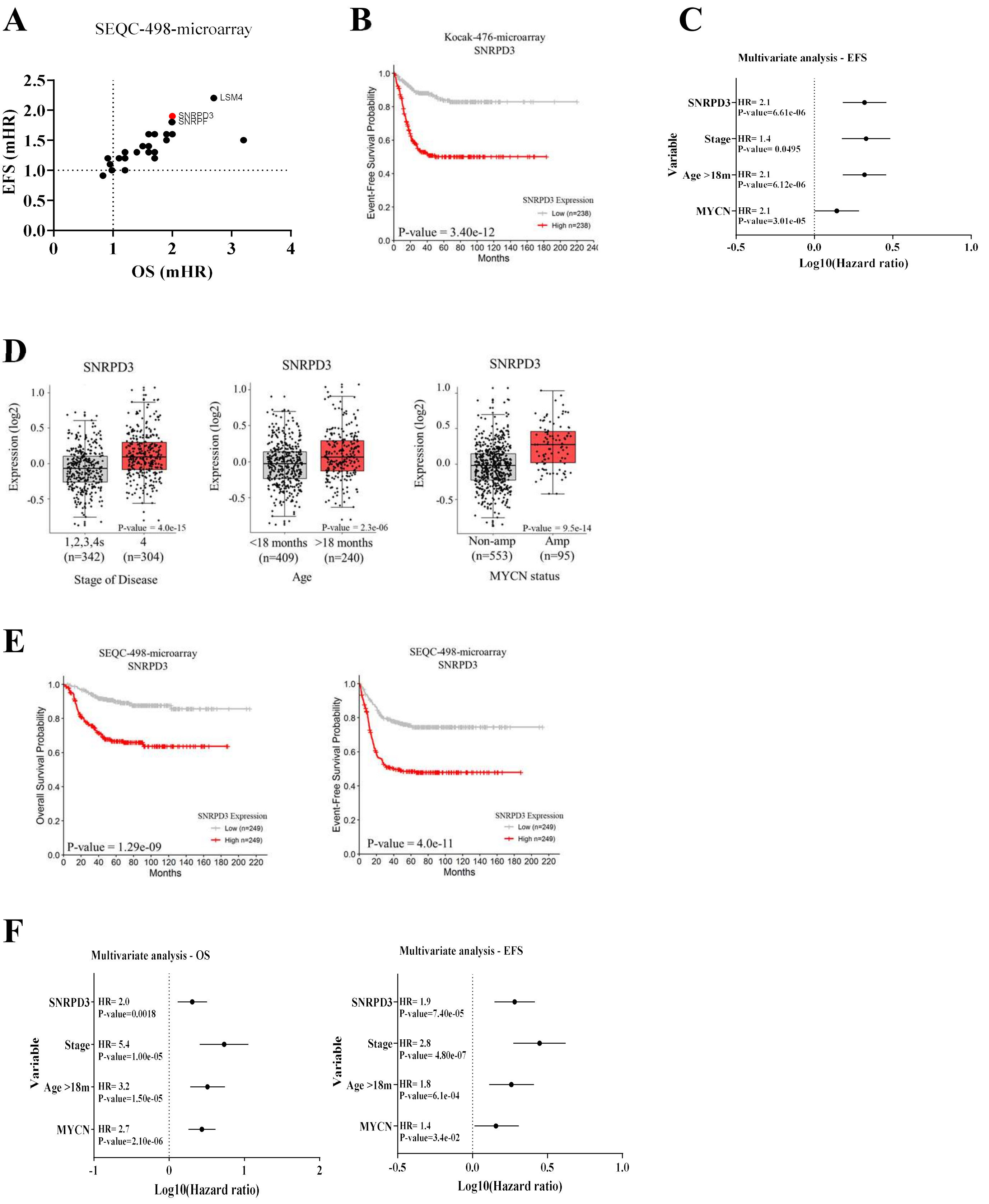

Supplementary figure 1 (Continued)

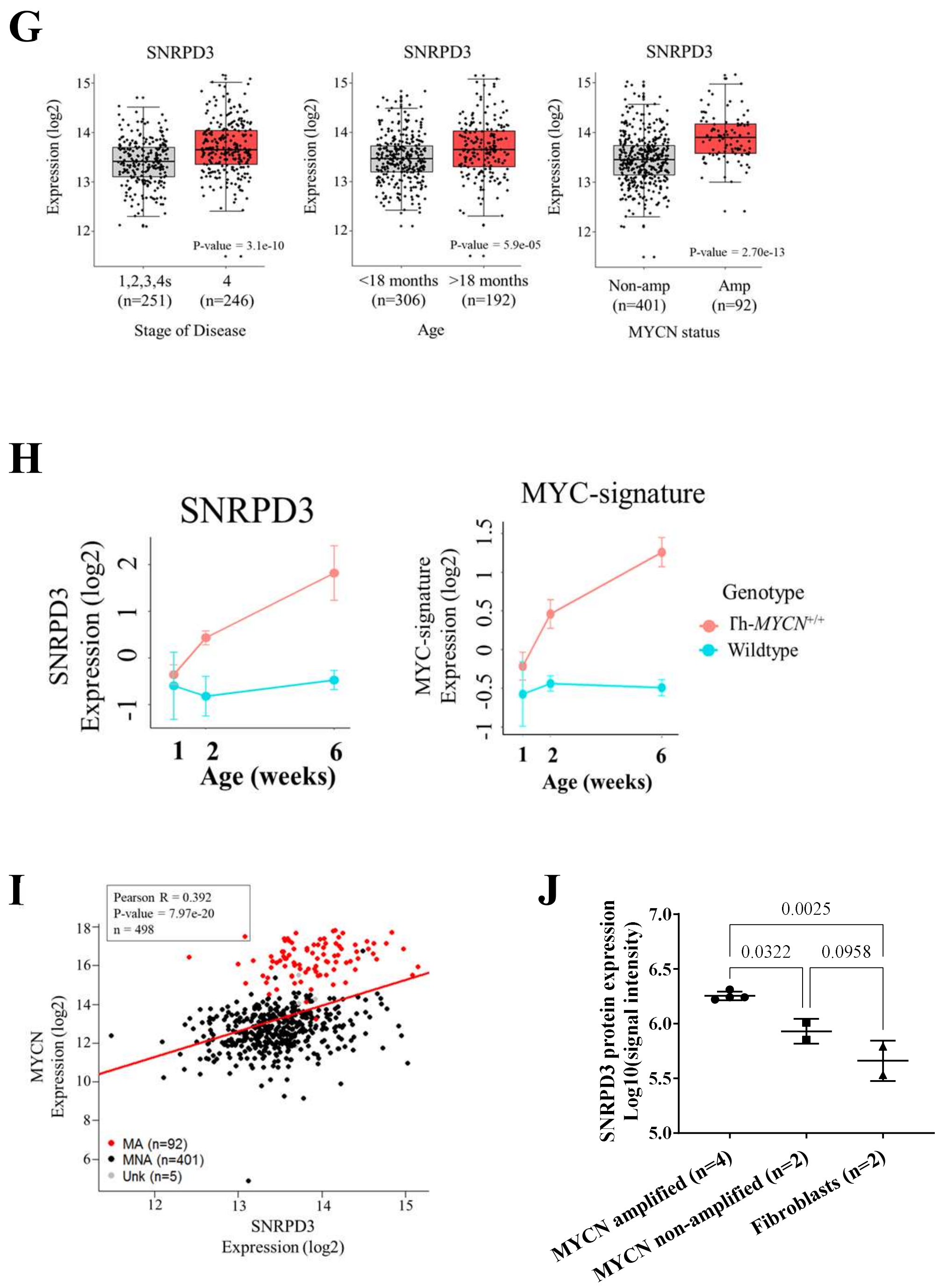

Supplementary figure 1. Some core snRNP assembly genes are up-regulated in neuroblastoma and are prognostic for patient outcomes (related to Fig. 1)

(**A**) Hazard ratios (HR) for overall survival (OS) and event-free survival (EFS) calculated by multivariate Cox proportional hazard (coxPH) analysis using the SEQC (n=498) neuroblastoma patient cohort of mRNAs from primary tumour tissue taken at diagnosis for the core spliceosome assembly genes (as defined by the GO term, GO:0000387). The dot plot represents the median HR (mHR) for EFS and OS from the CoxPH models for each gene. (**B**) Kaplan-Meier EFS curve obtained from the Kocak neuroblastoma patient mRNA sample cohort (n=476) dichotomised for the median *SNRPD3* expression. (**C**) EFS multivariate CoxPH analysis on the Kocak cohort (n=649) with high *SNRPD3* gene expression level compared with established neuroblastoma prognostic factors. Dots represent the median HR, whilst the lines represent the 95% confidence interval. (**D**) Box plots of *SNRPD3* expression subdivided using established neuroblastoma prognostic factors from the Kocak cohort. Patients were stratified by either stage of disease (stage 1, 2, 3, 4s vs 4), age at diagnosis (18 months of age), and *MYCN* status (*MYCN* non-amplified vs *MYCN*-amplified). (**E**) Kaplan-Meier OS and EFS curve obtained from the SEQC neuroblastoma patient cohort (n=498) dichotomised around the median SNRPD3 expression. (**F**) OS and EFS multivariate CoxPH analysis on the SEQC cohort with high SNRPD3 gene expression compared to established neuroblastoma prognostic factors. The dots represent the median HR, whilst lines represent the 95% confidence interval. (**G**) Box plots of *SNRPD3* expression subdivided using established neuroblastoma prognostic factors for the SEQC cohort. Patients were stratified by either stage of disease (stage 1, 2, 3, 4s vs 4), age at diagnosis (18 months of age), and *MYCN* status (*MYCN* non-amplified vs *MYCN*-amplified). (**H**) mRNA expression of *SNRPD3* and *MYC*-signature genes [29] in ganglia from Th-$MYCN^{+/+}$ mice compared to wildtype littermate mice, obtained at different postnatal ages (1, 2 and 6 weeks of age) transformed to a log2 expression scale. (**I**) Scatter plot with linear regression fit for the SEQC (n=498) neuroblastoma patient sample cohort assessing *MYCN* vs *SNRPD3* gene expression levels as a log2 expression scale. (**J**) A densitometric comparison of SNRPD3, MYCN, and c-MYC protein expression on immunoblotting from a range of *MYCN*-amplified (SK-N-BE(2)-C, CHP-134, KELLY, IMR-32), MYCN non-amplified (SK-N-AS, SH-SY5Y), and normal lung fibroblasts (MRC-5, WI-38) cells. Results represent n=3 independent biological replicates, mean ± SEM, where the P value was determined through a one-way ANOVA, with Tukey's multiple comparison tests.

Supplementary figure 2

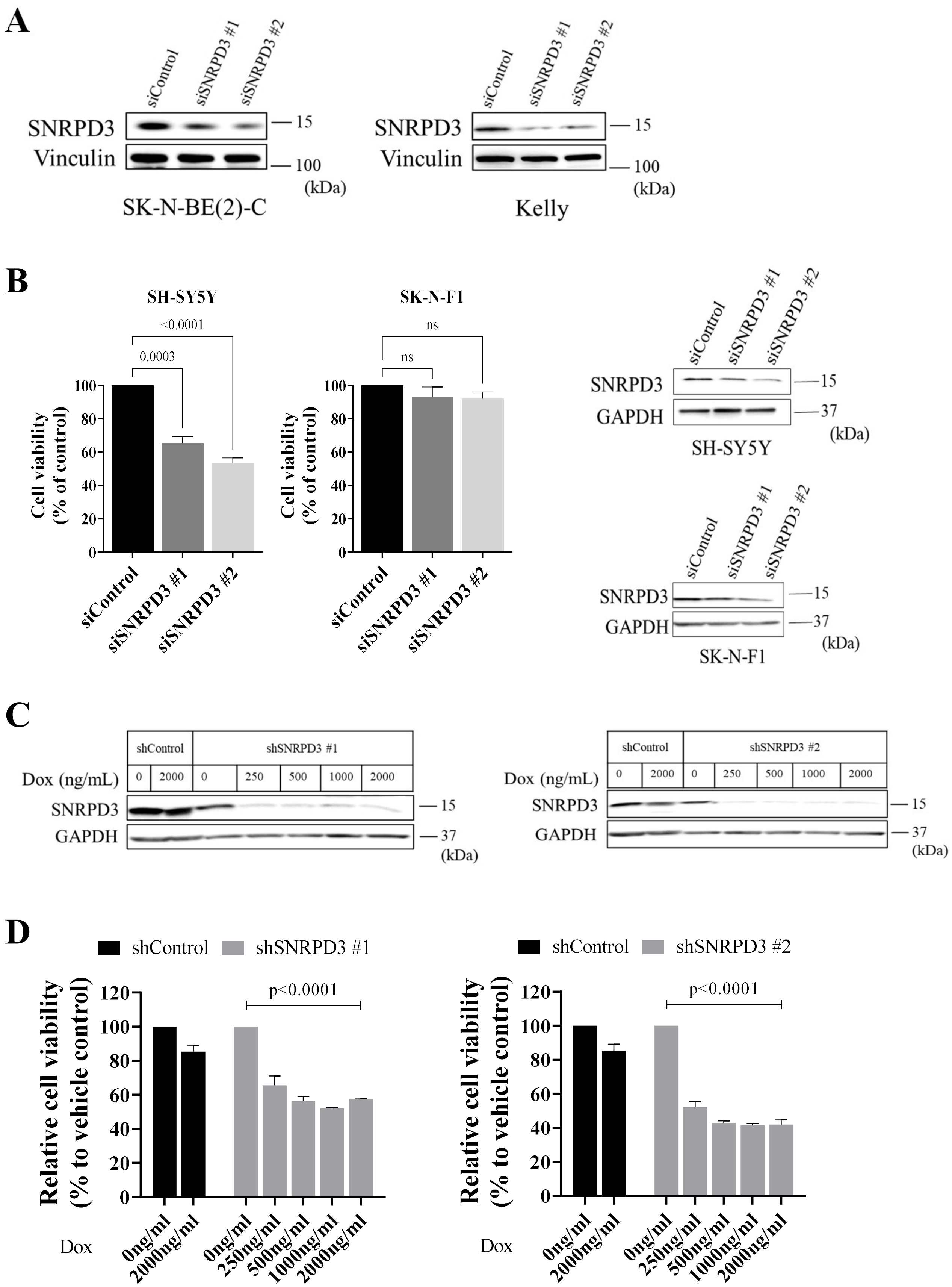

Supplementary figure 2 (Continued)

E

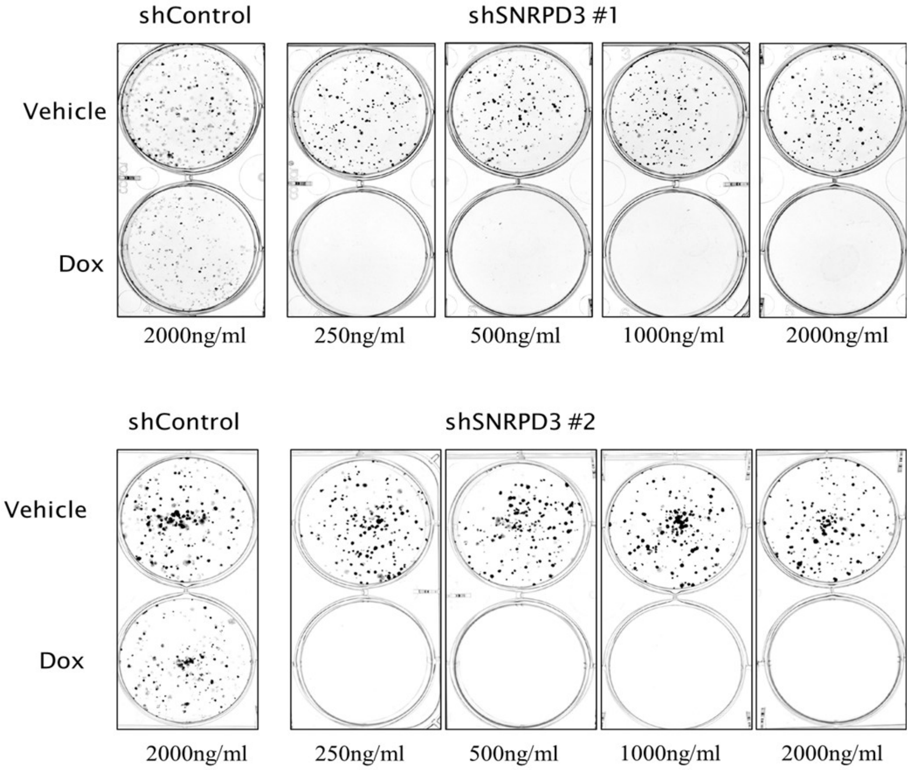

F

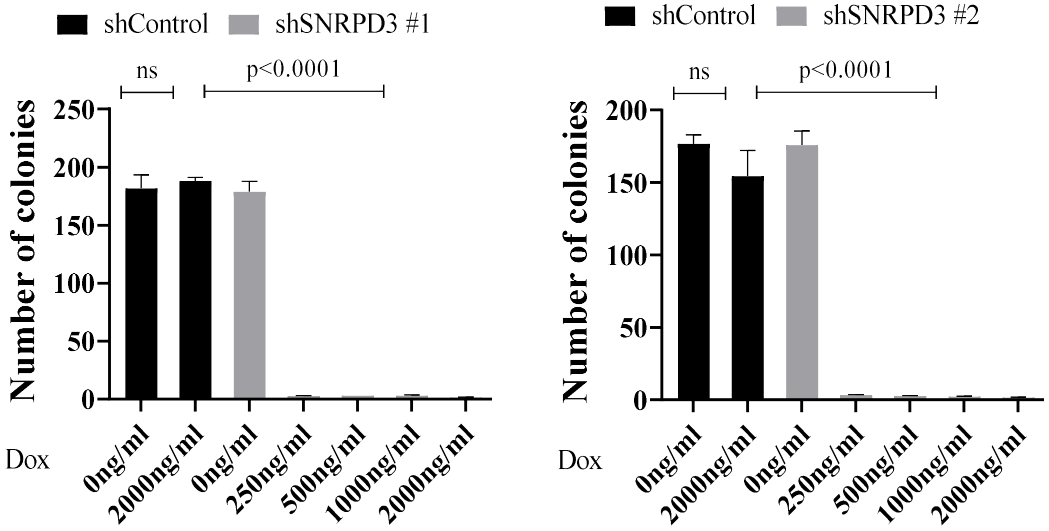

Supplementary figure 2. MYCN regulates the expression of the *SNRPD3* gene in neuroblastoma cells (related to Fig. 3)

(**A**) Representative western blot of total protein from SK-N-BE(2)-C and KELLY cells transfected with either control or SNRPD3 siRNA to confirm sufficient knockdown of SNRPD3 for the phenotypic assays. (**B**) SH-SY5Y and SK-N-F1 cells were transfected with siRNAs targeted against SNRPD3 or a control siRNA for 72 hours, then cell viability was measured. Differences in cell viability were compared with siControl. A representative western blot of total protein from SH-SY5Y and SK-N-F1 cells transfected with either control or SNRPD3 siRNA to confirm sufficient knockdown of SNRPD3 for the cell viability assay. Results represent n=3 independent biological replicates, mean ± SEM, where the P value was determined through a one-way ordinary ANOVA, with Dunnett multiple comparison testing. (**C**) A representative western blot of SNRPD3 expression in BE2C.shControl, BE2C.shSNRPD3 #1 and #2 cells following 72 hours treatment with increasing concentrations of doxycycline (0-2000 ng/mL). (**D**) Cell viability was measured 72 hours after BE2C.shControl, BE2C.shSNRPD3 #1 or #2 cells were treated with increasing concentrations of doxycycline (0-2000 ng/mL). (**E**) Five hundred BE2C.shControl, BE2C.shSNRPD3 #1 or #2 cells were treated with increasing concentrations of doxycycline (0-2000 ng/mL) and left for 10 days, followed by colony formation assays. (**F**) Quantification of the number of colonies, where the differences in colony formation were compared to the vehicle control. All Results represent n=3 independent biological replicates, mean ± SEM, where the P value was determined through a two-way ordinary ANOVA, with Tukey's multiple comparison testing, unless stated otherwise.

Supplementary figure 3

A

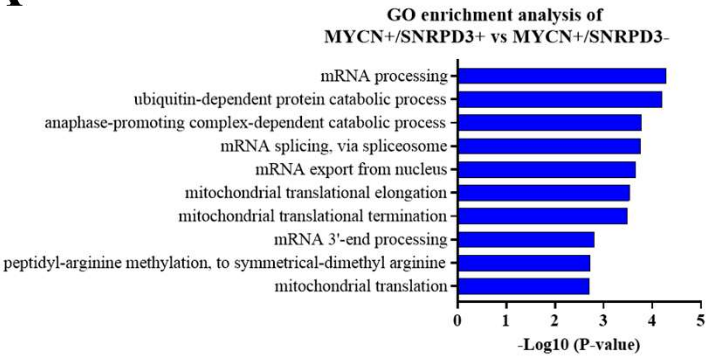

B

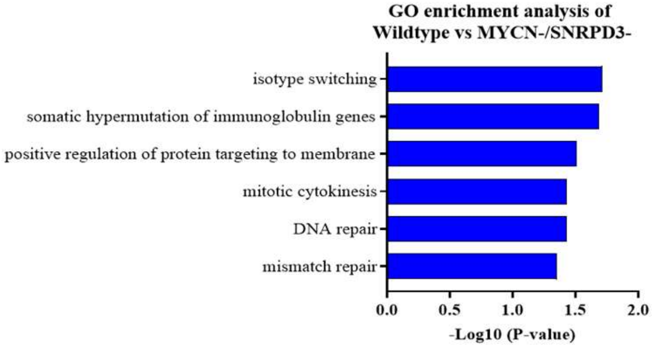

C

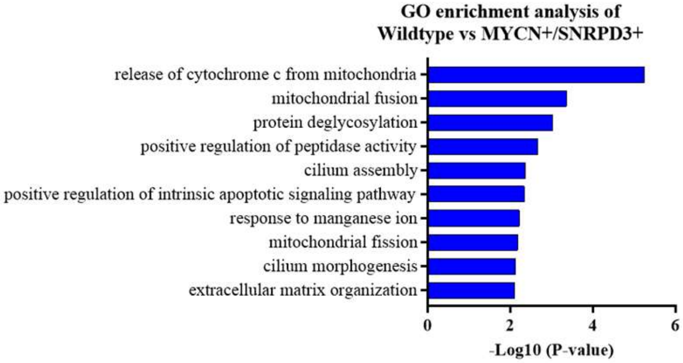

Supplementary figure 3 (Continued)

D

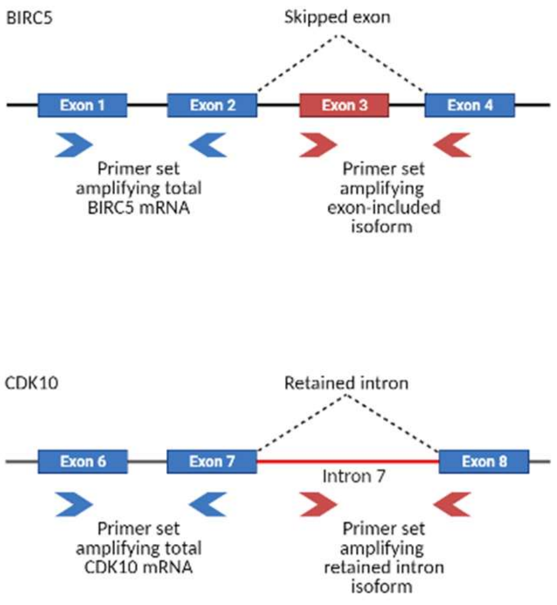

E

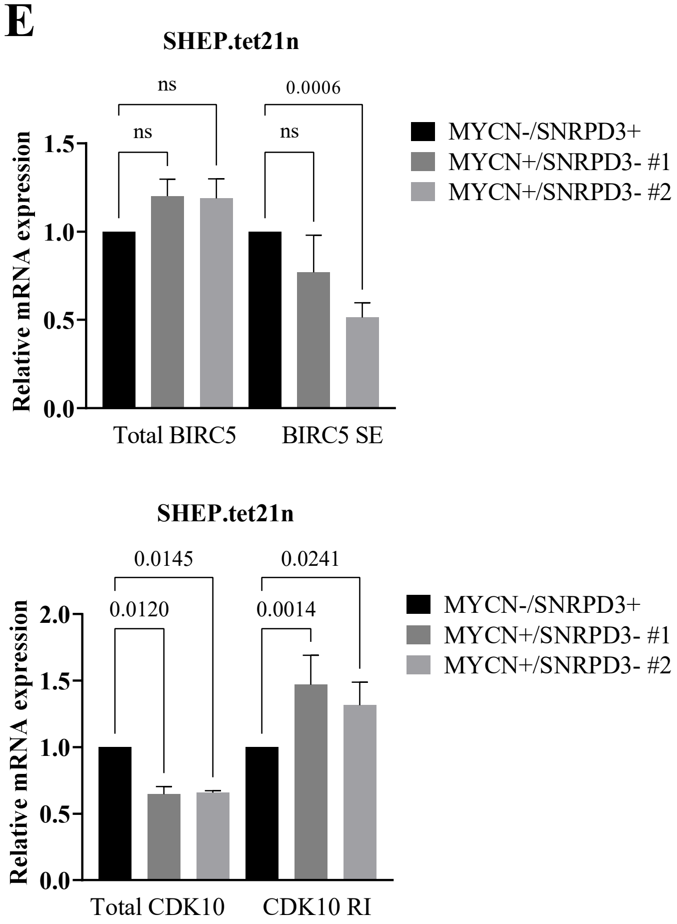

F

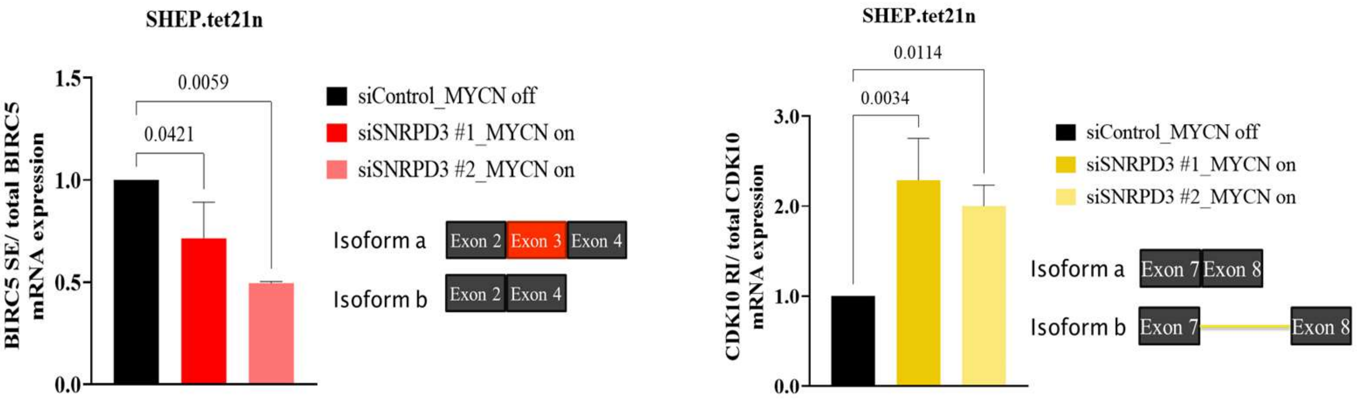

Supplementary figure 3. SNRPD3 alters the splicing of cell cycle genes in a MYCN-dependant manner (related to Fig. 4)

(**A**) Gene ontology showing enriched pathways in SHEP.tet21n cells treated without Dox (to induce MYCN) and transfected with control siRNA (MYCN+/SNRPD3+) compared to SHEP.tet21n cells treated without Dox and transfected with SNRPD3 siRNA (MYCN+/SNRPD3-). (**B**) Gene ontology showing enriched pathways in SHEP.tet21n cells treated with Dox (to repress MYCN) and transfected with control siRNA (Wildtype; MYCN-/SNRPD3+) compared to SHEP.tet21n cells treated with Dox and transfected with SNRPD3 siRNA (MYCN-/SNRPD3-). (**C**) Gene ontology showing enriched pathways in SHEP.tet21n cells treated with Dox and transfected with control siRNA (wildtype; MYCN-/SNRPD3+) compared to SHEP.tet21n cells treated without Dox and transfected with control siRNA (MYCN+/SNRPD3+). (**D**) Schematic of splicing primer design for total and isoform specific BIRC5 and CDK10. (**E**) RT-qPCR of total and isoform specific BIRC5 (BIRC5 SE) and CDK10 (CDK10 RI) was performed on SHEP.tet21n cells treated with doxycycline and transfected with control siRNA (wildtype; MYCN-/SNRPD3+) and SHEP.tet21n cells treated without doxycycline and transfected with SNRPD3 siRNA#1 and #2 (MYCN+/SNRPD3-). (**F**) Bar graph of RT-qPCR analysis of the expression of BIRC5 with SE/BIRC5 total or CDK10 with RI/CDK10 in SHEP.tet21n cells, where isoform b depicts exon 3 skipping for BIRC5 and intron 7 retention for CDK10. All Results represent n=3 independent biological replicates, mean ± SEM, where the P value was determined through a two-way ordinary ANOVA, with Tukey's multiple comparison testing.

Supplementary figure 4

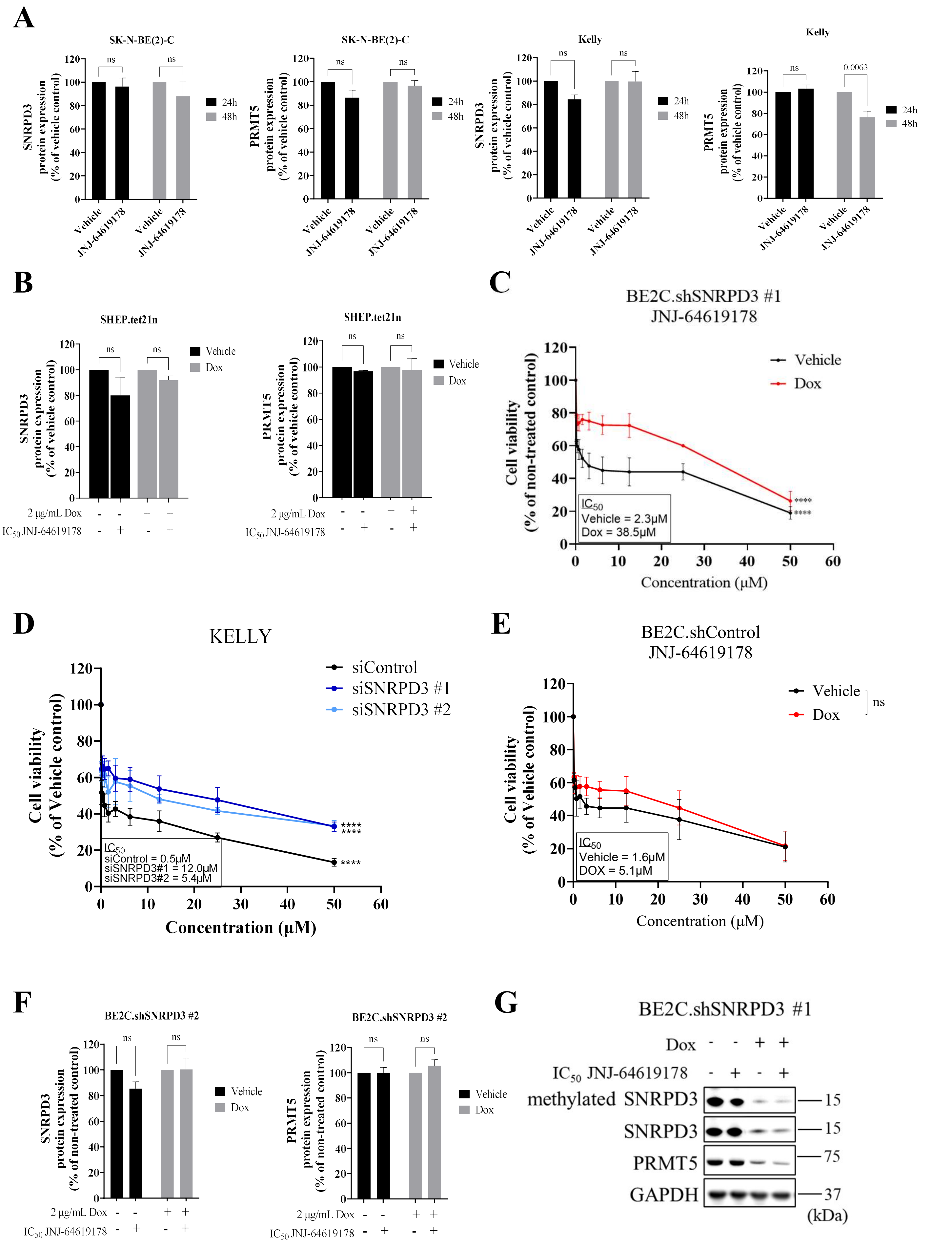

Supplementary figure 4 (Continued)

H

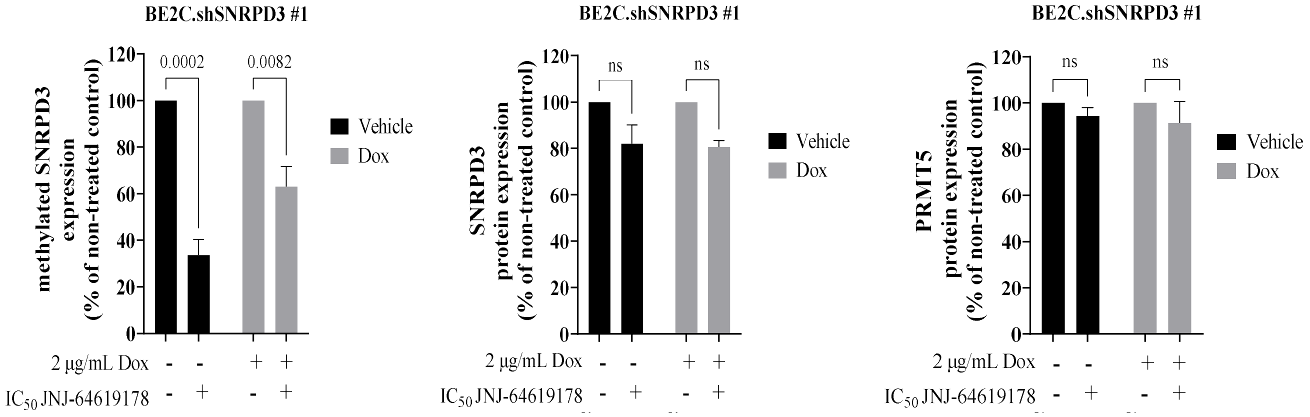

I

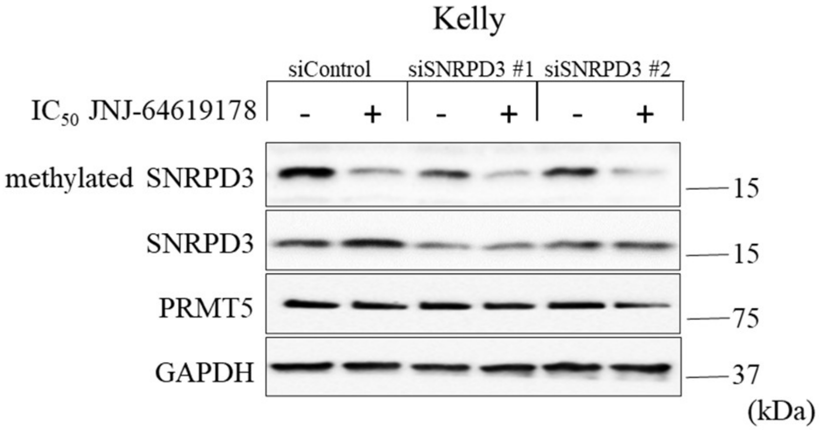

J

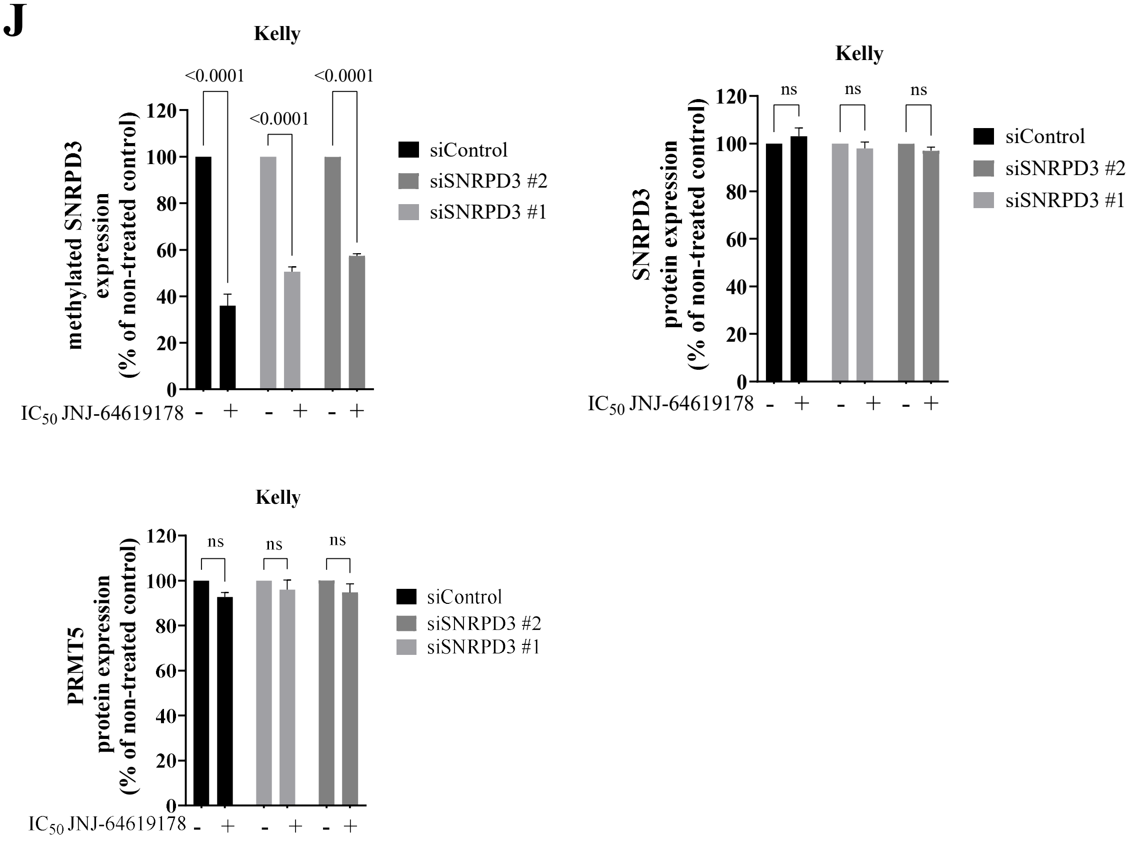

Supplementary figure 4 (Continued)

K

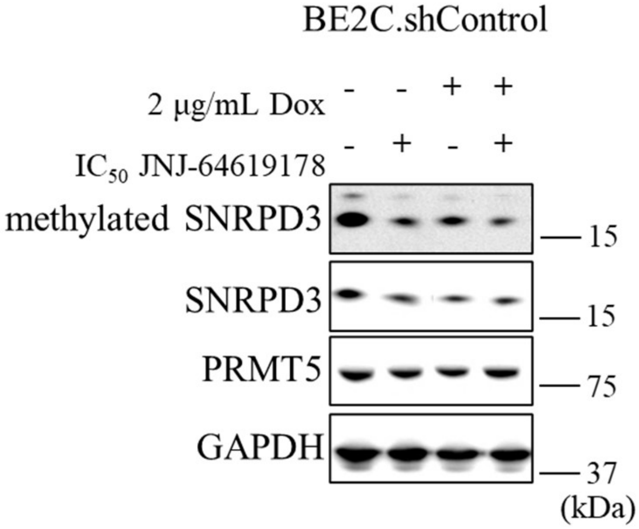

L

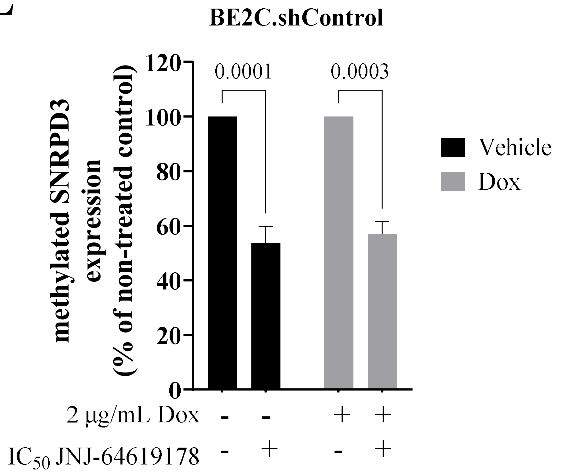

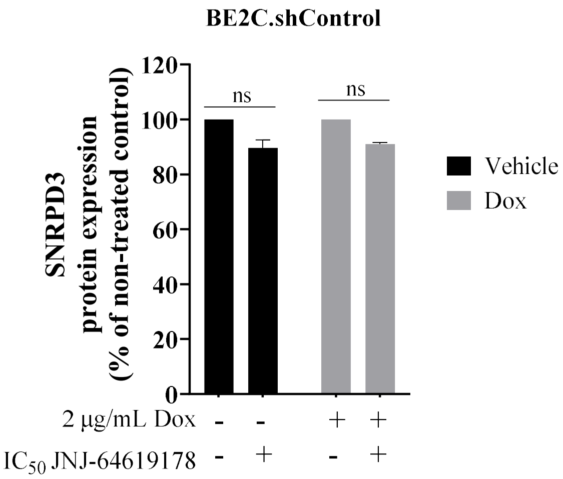

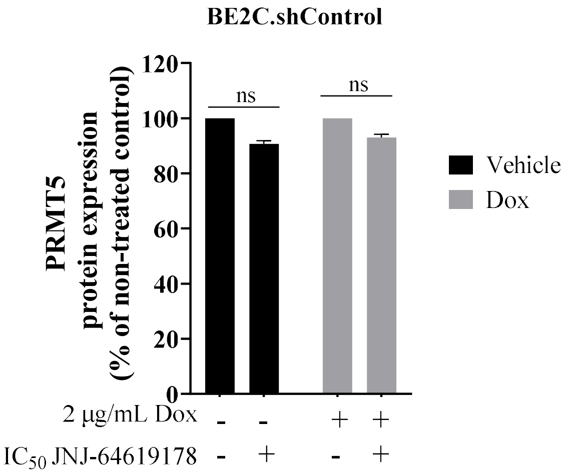

Supplementary figure 4. Chemical inhibition of PRMT5 has selective toxicity for neuroblastoma cells compared to normal myofibroblast cells (related to Fig. 6)

(**A**) Densitometry analysis of SNRPD3 and PRMT5 expression in SK-N-BE(2)-C and KELLY cells treated with IC_{50} concentrations of the PRMT5 inhibitor, JNJ-64619178, at 24 and 48 hours, where difference in protein expression was compared to vehicle control. (**B**) Densitometry analysis of SNRPD3 and PRMT5 expression in SHEP.tet21n cells treated with vehicle or Dox followed by treatment with IC_{50} concentrations of JNJ-64619178 at 24 hours, where difference in protein expression was compared to vehicle control. (**C**) Cell viability was measured 72 hours after SK-N-BE(2)-C.shSNRPD3 #1 cells, treated with either vehicle or Dox, were also treated with increasing concentrations (0-50μM) of JNJ-64619178. (**D**) Cell viability was measured 72 hours after control or SNRPD3 siRNA transfected KELLY cells, were treated with increasing concentrations (0-50μM) of JNJ-64619178. (**E**) Cell viability was measured 72 hours after vehicle or Dox treated BE2C.shControl cells were treated with increasing concentrations (0-50μM) of JNJ-64619178. (**F**) Densitometry analysis of SNRPD3 and PRMT5 expression in SK-N-BE(2)-C.shSNRPD3 #2 cells treated with vehicle or Dox followed by treatment with IC_{50} concentrations of JNJ-64619178 at 24 hours, where difference in protein expression was compared to vehicle control. (**G**) Representative western blot of SNRPD3 protein arginine methylation, SNRPD3, and PRMT5 expression following treatment with IC_{50} concentration of JNJ-64619178 in SK-N-BE(2)-C.shSNRPD3 #1 cells for 24 hours, followed by densitometry analysis, where difference in protein expression was compared to vehicle control. (**H**) Densitometry analysis of SNRPD3 protein arginine methylation, SNRPD3, and PRMT5 expression in SK-N-BE(2)-C.shSNRPD3 #1 cells treated with vehicle or Dox followed by treatment with IC_{50} concentrations of JNJ-64619178 at 24 hours, where difference in protein expression was compared to vehicle control. (**I**) Representative western blot of methylated SNRPD3, SNRPD3, and PRMT5 expression following treatment with IC50 concentration of JNJ-64619178 in control or SNRPD3 siRNA transfected KELLY cells for 24 hours. (**J**) Densitometry analysis of methylated SNRPD3, SNRPD3 and PRMT5 expression in control or SNRPD3 siRNA transfected KELLY cells treated with IC50 concentrations of JNJ-64619178 at 24 hours, where difference in protein expression was compared to vehicle control. (**K**) Representative western blot of methylated SNRPD3, SNRPD3, and PRMT5 expression following treatment with IC50 concentration of JNJ-64619178 in vehicle or Dox treated BE2C.shControl cells for 24 hours.

Supplementary figure 4. Chemical inhibition of PRMT5 has selective toxicity for neuroblastoma cells compared to normal myofibroblast cells (related to Fig. 6) (Continued) (**L**) Densitometry analysis of methylated SNRPD3, SNRPD3, and PRMT5 expression in vehicle or Dox treated BE2C.shControl cells treated with IC50 concentrations of JNJ-64619178 at 24 hours, where difference in protein expression was compared to vehicle control. Two-way ANOVA statistical test was performed for each concentration compared back to no drug control (at 0 μM) (****$p<0.0001$). All results represent n=3 independent biological replicates, mean ± SEM, where the P value was determined through a two-way ANOVA, with Tukey's or Dunnett multiple comparison tests.

Supplementary Table 1 – List of primer sequences used

PRIMER SEQUENCES		
Gene	Primer sequence	
Primer sequences for gene expression analysis		
SNRPD3	fwd	5'-AAAGTAGGCCAGAGCCGAAC-3'
	rev	5'-TGGACATCTGGCAGTTCATGT-3'
MYCN	fwd	5'-CGACCACAAGGCCCTCAGTA-3'
	rev	5'-CAGCCTTGGTGTTGGAGGAG-3'
GAPDH	fwd	5'-TCGGAGTCAACGGATTTGGT-3'
	rev	5'-TTCCCGTTCTCAGCCTTGAC-3'
Primer sequences for chromatin immunoprecipitation		
Negative control	fwd	5'-AGGCACTTAGATATGCTTGG-3'
	rev	5'-AAAGGCATGGACAGTCATAG-3'
SNRPD3 (BE2C)	fwd	5'-TGCTTGGAAGTGTGAGCACC-3'
	rev	5'-AAGGCGTGAGTCAAGCGAA-3'
SNRPD3 (Kelly)	fwd	5'-ACTGGTGCCCTAGTTTCCCA-3'
	rev	5'-GAAAGATGCTACGGCGAAGG-3'
Primer sequences for alternative splicing analysis		
BIRC5 total	fwd	5'-ACCGCATCTCTACATTCAAG-3'
	rev	5'-TTTTATGTTCCTCTATGGGGT-3'
BIRC5 SE	fwd	5'-GTAATGCAGTTCTGGTAACG-3'
	rev	5'-ACATTGAACAGGGTTTGAGC-3'
CDK10 total	fwd	5'-CTTCATTATCCACAGGGACC-3'
	rev	5'-CCTTTTCTTAGGGTCGTACA-3'
CDK10 RI	fwd	5'-CCTAGATGGCACTTGGTGA-3'
	rev	5'-TTACCAGAGAGTGACCACC-3'
GAPDH	fwd	5'-TCGGAGTCAACGGATTTGGT-3'
	rev	5'-TTCCCGTTCTCAGCCTTGAC-3'

Supplementary Table 2 - Topmost significant differentially spliced cell cycle genes identified from rMATS analysis from the Wildtype vs MYCN+/SNRPD3- comparison

Gene Symbol	PValue	FDR	deltaPSI
BIRC5	2.22045E-16	1.15695E-13	-0.158
CDK10	2.88658E-15	7.03646E-13	0.200
CHFR	1.72085E-14	9.8876E-12	-0.077
ANAPC16	1.15796E-13	5.275E-11	-0.107
TERF1	2.32259E-13	9.98372E-11	-0.212
CCNB1	7.73492E-13	2.85296E-10	-0.021
CDK2	1.8695E-12	6.19597E-10	-0.113
SUGT1	2.67586E-12	8.71117E-10	-0.031
SPDL1	6.36902E-12	1.89646E-09	0.116
CENPW	1.10837E-11	1.76096E-09	0.052
AURKB	1.12759E-11	3.14462E-09	-0.039
SPDL1	1.39337E-11	3.80238E-09	0.121
AURKB	4.31819E-11	1.06871E-08	-0.085
NSL1	4.46757E-11	1.1033E-08	-0.039
ZNF207	1.48268E-10	3.24151E-08	-0.074

Supplementary Table 3 - IC_{50} concentrations from treatment with JNJ-64619178 for 72 hours in neuroblastoma and normal lung fibroblast cell lines

Cell line		IC_{50} (µM)
		JNJ-64619178
SK-N-BE(2)-C	MYCN-amplified	3.296
KELLY	MYCN-amplified	3.912
SK-N-AS	MYCN non-amplified	11.82
SK-N-F1	MYCN non-amplified	194.5
MRC-5	Normal	84.59
